# Systems Biomedicine of Primary and Metastatic Colorectal Cancer Reveals Potential Therapeutic Targets

**DOI:** 10.3389/fonc.2021.597536

**Published:** 2021-06-24

**Authors:** Mehran Piran, Neda Sepahi, Afagh Moattari, Amir Rahimi, Ali Ghanbariasad

**Affiliations:** ^1^ Department of Anatomy and Developmental Biology, Monash University, Melbourne, VIC, Australia; ^2^ Department of Bacteriology and Virology, Medical School, Shiraz University of Medical Sciences, Shiraz, Iran; ^3^ Noncommunicable Diseases Research Center, Fasa University of Medical Sciences, Fasa, Iran; ^4^ Bioinformatics and Computational Biology Research Center, Shiraz University of Medical Sciences, Shiraz, Iran

**Keywords:** colorectal cancer, cancer progression, EMT, metastasis, therapeutic/curative targets, diagnostic biomarkers

## Abstract

Colorectal cancer (CRC) is one of the major causes of cancer deaths across the world. Patients’ survival at time of diagnosis depends mainly on stage of the tumor. Therefore, understanding the molecular mechanisms from low-grade to high-grade stages of cancer that lead to cellular migration from one tissue/organ to another tissue/organ is essential for implementing therapeutic approaches. To this end, we performed a unique meta-analysis flowchart by identifying differentially expressed genes (DEGs) between normal, primary (primary sites), and metastatic samples (Colorectal metastatic lesions in liver and lung) in some Test datasets. DEGs were employed to construct a protein-protein interaction (PPI) network. A smaller network containing 39 DEGs was then extracted from the PPI network whose nodes expression induction or suppression alone or in combination with each other would inhibit tumor progression or metastasis. These DEGs were then verified by gene expression profiling, survival analysis, and multiple Validation datasets. We suggested for the first time that downregulation of mitochondrial genes, including ETHE1, SQOR, TST, and GPX3, would help colorectal cancer cells to produce more energy under hypoxic conditions through mechanisms that are different from “Warburg Effect”. Augmentation of given antioxidants and repression of P4HA1 and COL1A2 genes could be a choice of CRC treatment. Moreover, promoting active GSK-3β together with expression control of EIF2B would prevent EMT. We also proposed that OAS1 expression enhancement can induce the anti-cancer effects of interferon-gamma, while suppression of CTSH hinders formation of focal adhesions. ATF5 expression suppression sensitizes cancer cells to anchorage-dependent death signals, while LGALS4 induction recovers cell-cell junctions. These inhibitions and inductions would be another combinatory mechanism that inhibits EMT and cell migration. Furthermore, expression inhibition of TMPO, TOP2A, RFC3, GINS1, and CKS2 genes could prevent tumor growth. Besides, TRIB3 suppression would be a promising target for anti−angiogenic therapy. SORD is a poorly studied enzyme in cancer, found to be upregulated in CRC. Finally, TMEM131 and DARS genes were identified in this study whose roles have never been interrogated in any kind of cancer, neither as a biomarker nor curative target. All the mentioned mechanisms must be further validated by experimental wet-lab techniques.

## Introduction

Colorectal cancer (CRC) is a major global medical burden worldwide (1). Approximately more than one million people are diagnosed with CRC each year, and about half of them die of CRC annually (2). Complex genetic interactions are combined with environmental factors to trigger a cell to become cancerous. Among them, aberrant growth factor signals contribute to uncontrolled cells’ proliferation, which ultimately leads to metastasis. Contrary to early-stage tumor cells, malignant cells have the ability to detach from the stroma as well as acquire motility (3). This event happens during a process called Epithelial-Mesenchymal Transition (EMT), in which cells lose their epithelial characteristic, including adhesion, and subsequently dedifferentiate into mesenchymal mobile cells (4). Therefore, Investigating DEGs between primary and metastatic sites of tumors would aim to recognize key factors playing roles in cell migration. We performed the statistical analysis between primary sites and metastatic sites in one part of the analyses. While primary sites were non-malignant colon biopsies in Test datasets, CRC metastatic sites were located on the other organs.

Many molecular and pathway targets have been identified for treatment of CRC during the past decades. Besides, growing progresses have been made in development of chemotherapy and antibody drugs (5). Tyrosine kinase (TK) targeting monoclonal antibodies and small-molecule tyrosine kinase inhibitors are effective strategies (6). Targeting cancer-related inflammation biomarkers like IL-6/JAK/STAT3 pathway, which inhibits progression of solid tumors, is another beneficial therapeutic strategy (7). In addition, restraint of cytosolic β‐catenin *via* disturbing hyperactive Wnt/β‐catenin signaling pathway could be another treatment approach for colorectal and many other types of cancer (8–10). Inhibition of matrix metalloproteinases (MMPs) and TGFβ signaling pathways is a therapeutic approach to prevent liver metastasis (11–14). Furthermore, PI3K inhibition suppresses lung metastasis in CRC patients (15, 16). Among the known anticancer drugs, Cetuximab is one of the popular ones, which is a monoclonal antibody against epidermal growth factor receptor (EGFR) (17). Furthermore, vascular endothelial growth factor (VEGF) antibody, bevacizumab, is the standard treatment for metastatic colorectal cancer (18).

**Figure 1:**
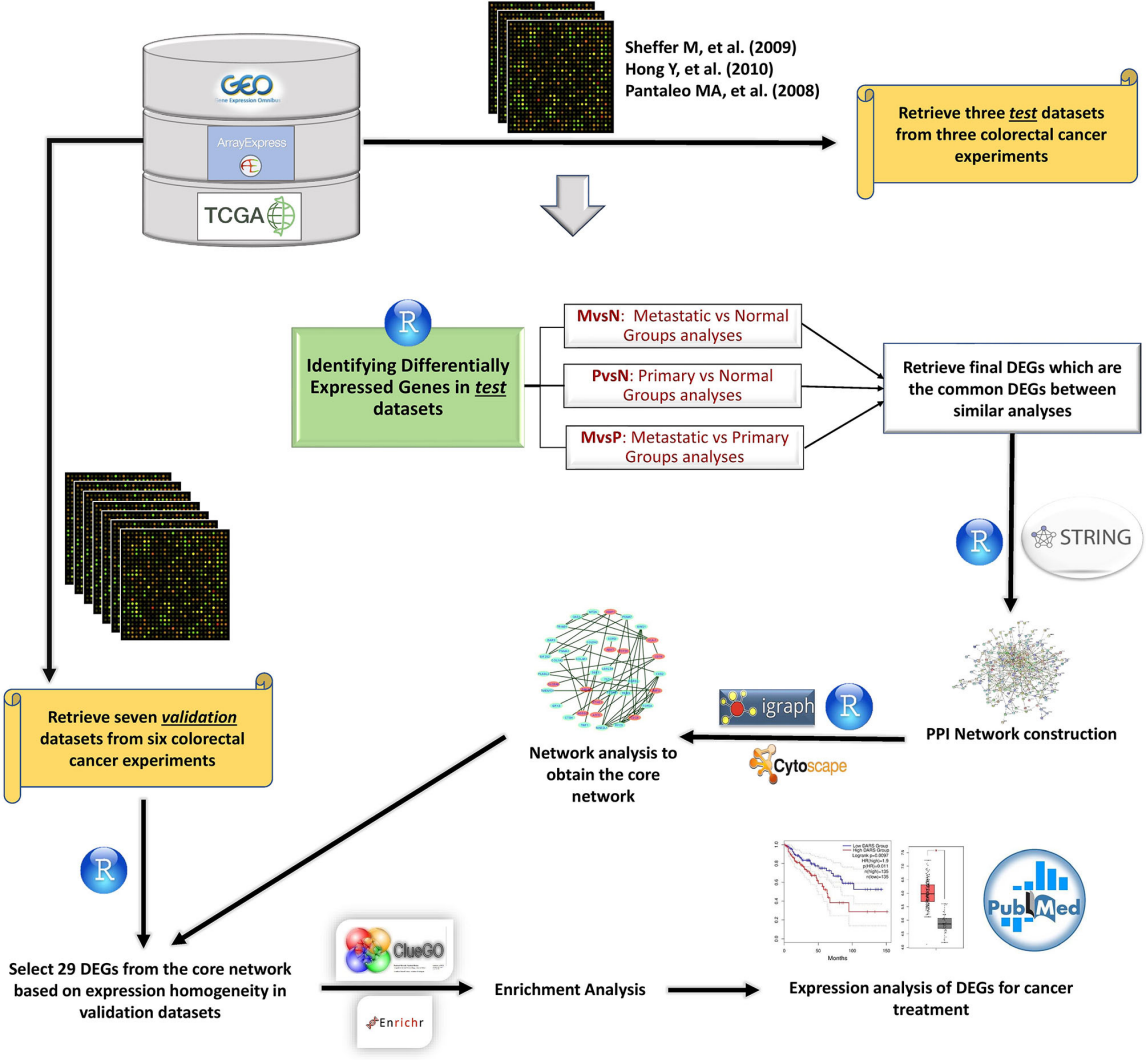
The meta-analysis flowchart to attain therapeutic genetic targets. Gene expression datasets were extracted from different databases. Data were analyzed and visualized using R programming language. DEGs were obtained from analyzing Test datasets, then verified by Validation datasets. STRING database was utilized to construct the PPI network from DEGs. R software was used to analyze the network. Cytoscape was employed to visualize the networks, and enrichment results were obtained from ClueGO Cytoscape plugin and Enrichr online tools. Next, survival analysis and expression profiling were used for more validation of expression results. Finally, our results were compared to other studies, and molecular mechanism of validated DEGs was interrogated to propose a combination of target therapies.

The aim of this study was to suggest multiple combinations of genetic targets that can prevent cancer progression. Therefore, We looked for the unknown key factors that partially control one or more steps of cancer progression, including cell proliferation, transformation, angiogenesis, and metastasis to the distant secondary sites. One way to identify molecular mechanism of pathogenesis in a biological context is to analyze transcriptomic data. Systematic investigation of gene expression data and cellular and molecular information in the literature for identified DEGs in normal and cancer tissues helped us to propose a number of these DEGs as therapeutic genetic targets. Once these targets were identified, we would see which ones can be targeted together in order to hinder cancer progression because each of the proposed genetic targets could control to some extent different steps of tumor formation towards malignancy. We conducted a unique meta-analysis flowchart where we separated datasets into two sets of Test and Validation datasets in order to not only recognize DE genes but also introduce them as the curative CRC targets. Furthermore, the shortest pathway scoring system for neighborhood finding around the core genes was first introduced by Seth I. Berger and his colleagues (Systems Pharmacology of Arrhythmias) on Long-QT syndrome (LQTS) PPI Network (19). They realized that the neighborhood ranking around the twelve core genes (Drivers of the LQTS Syndrome) discovered the genes that are targeted with FDA-approved drugs. Since our goal was to find therapeutic genetic targets to inhibit metastasis, we applied the algorithm on colorectal cancer PPI network for the first time. First of all, three Test datasets were constructed from three microarray studies, and DE genes were excavated for any pairwise comparison between four groups of samples. Common DEGs between similar analyses in Test datasets were regarded as final DEGs employed for PPI network assembly. Test datasets provided us with a sufficient number of common DEGs with desired log-fold change and p-value thresholds for network construction. Twelve common genes called Core genes were recognized that their expression were different between primary and metastatic sites. A smaller network called Core network was then extracted from the PPI based on a shortest-path-based scoring formula on these Core genes. To compensate for the small number of datasets in Test set, seven Validation datasets were employed from different genomic repositories to validate selected DEGs in the Core network (**Figure 1**). Besides, expression profiling and survival analysis provided more evidence about the accuracy of our results. We obtained some DEGs involved in cancer progression whose expression could be targeted (suppressed or induced) individually or in combination with one another for CRC treatment. Moreover, some of those gene expressions were proposed to be CRC biomarkers.

## Materials and Methods

### Database Searching and Recognizing Pertinent Experiments

Gene Expression Omnibus (GEO) (http://www.ncbi.nlm.nih.gov/geo/) and ArrayExpress (https://www.ebi.ac.uk/arrayexpress/) repositories were searched to detect experiments containing high-quality transcriptomic samples concordance to the study design. Colorectal/colon cancer, primary, EMT, and metastasis were the keywords utilized in the search, but search was filtered for Homo sapiens. Microarray raw data (.CEL files) for GSE41258, GSE9348, and GSE10961 studies were downloaded from GEO and ArrayExpress to create Test datasets (20–22). Dataset1 and Dataset2 were constructed from samples in GSE41258 study. Dataset1 encompassed CRC liver metastasis samples, primary samples and normal samples, while Dataset2 contained CRC lung metastasis samples, primary samples and normal samples. To construct Dataset3, normal samples and primary samples were extracted from GSE9348 study, but colorectal liver metastasis samples were obtained from GSE10961 study. In all datasets, normal samples were healthy colon tissues adjacent to the primary tumors and primary samples were non-metastatic colorectal biopsies. To make Validation datasets, two new datasets were constructed from GSE41258 study whose samples were not present in Test datasets. One dataset contained CRC liver metastasis samples, and another one contained CRC lung metastasis samples. In addition, a dataset was constructed from count RNAseq files in The Cancer Genome Atlas (TCGA) database (TCGA dataset). Three RNAseq datasets were constructed from GSE50760, GSE144259, and GSE89393 studies encompassing CRC liver metastasis samples (23–25). The last dataset was built from GSE40367 microarray study containing CRC liver metastasis samples (26). Except for TCGA dataset, all Test and Validation datasets contained three groups of metastatic, primary and normal samples.

### Identifying Differential Expressed Genes in Microarray Datasets

R programming language (v3.6.2) was used to import and analyze data for each dataset separately. Preprocessing step involving background correction and probe summarization was done using RMA method in “affy” package (27). Absent probesets were identified using “mas5calls” function in this package. If a probeset contained more than two absent values in each group of samples, that probeset was regarded as absent and removed from the expression matrix. Besides, outlier samples were identified and removed using PCA and hierarchical clustering methods. Next, data were normalized using Quantile normalization approach (28). Then, standard deviation (SD) for each gene was computed, and median of all SDs was utilized as a cutoff to remove low-variant probesets. Therefore, low-variant probesets no longer influenced the significance of the high-variant genes. “Many to Many” problem (29), which is mapping multiple probesets to the same gene symbol, was solved using “nsFilter” function in “genefilter” package (30). This function selects the probeset with the highest Interquartile range (IQR) to map to the gene symbol. “limma” R package, which applies linear models on the expression matrix, was utilized to discover DE genes between all groups of samples (31). Genes with absolute log fold change larger than 0.5 and Benjamini-Hochberg adjusted p-value (32) less than 0.05 were selected as the DEGs.

### Identifying Differential Expressed Genes in RNAseq Datasets

Count files for five primary samples containing more than 90 percent tumor cells as well as five normal samples involving 100 percent normal cells were downloaded from TCGA database. Each sample was imported into R, and they were merged together to construct the TCGA expression matrix encompassing a five-sample primary group and a five-sample normal group. Genes with zero expressions in the two groups were omitted. Then, data were normalized with “DESeq2” R package (33), and DEGs were identified between the two groups. For RNAseq datasets in GEO, FPKM normalized data were downloaded and imported into R. data were log2 transformed, and using “limma” R package, DEGs were identified between the groups.

### Network Construction

Final DEGs were imported into STGRING web server database and different sources of evidence were chosen to generate interactions and the Protein-Protein Interaction (PPI) network. Afterward, Interactions were downloaded and imported into R programming language in form of an annotated edgelist. Next, extra information were removed, and an undirected edgelist was obtained using “igraph” R package (34). Interaction scores were considered as weights, so a weighted PPI network was created. The giant component of the weighted PPI network was then extracted for further analyses. The weighted adjacency matrix of the giant component was created and transformed into a symmetric matrix. It was then modified into a new adjacency matrix using topological overlapping measure (TOM) function in “WGCNA” R package (35). Finally, this modified adjacency matrix was subtracted from one to create a distance adjacency matrix.

### Neighborhood Ranking to the Core Genes

Using Dijkstra algorithm in R, a matrix of all shortest paths, called SP, between all pairs of nodes was constructed from the distance adjacency matrix (36). By utilizing this matrix, a distance score, DJ, for each node in the PPI network was computed. Moreover, we considered DEGs between metastatic versus primary analysis as the Core nodes in the PPI network. Dj is a scoring formula that is the average of the shortest paths from all the non-core nodes to reach the node j subtracted from the average of the shortest paths from the Core nodes to reach the node j divided by the average of the all shortest paths to reach the node j from the whole network. This scoring system implies how much close each node is to the Core nodes (19, 37).

$${\text{D}}_{\text{j}}=\Sigma_{\text{i}\notin \text{c}}\text{S}{\text{P}}_{\text{i j}}/\text{N C}-\Sigma_{\text{i}\in \text{c}}\text{S}{\text{P}}_{\text{i j}}/\text{C}/\Sigma_{\text{i}}\text{S}{\text{P}}_{\text{i j}}/\text{N C}+\text{C}$$

C is the number of Core nodes, and NC is the number of non-core nodes. ∑_i_ ∉ c SP_ij_ is the sum of all distances in SP matrix between node j and all the non-core nodes. ∑_i_ ∈ c SP_ij_ is the sum of the distances between node j and all the Core nodes. ∑_i_ SP_ij_ is the sum of the distances between node j and all the nodes. A positive score implies that node j is closer to the Core nodes than the rest of the nodes. Nodes with positive scores were kept, but the others were removed from the network. It should be noted that D scores were calculated without imposing any threshold on edge weights. The R source codes for the network analysis are available at https://github.com/mehranpiran/Meta-Analysis.

### Enrichment Analysis

Enrichment analysis was performed using ClueGO Cytoscape plugin (38). Enriched terms for biological processes were obtained from GO repository. For pathway enrichment analysis, information in KEGG (39), Reactome (40) and WikiPathways (41) databases were used. P-value were adjusted using Benjamini-Hochberg method and cut off was set on 0.05. In addition to Cytoscape, Enrichment analysis was performed using Enrichr online tool (42) as well. Enriched terms for biological processes were obtained from GO repository. For pathway enrichment analysis, WikiPathways signaling repository version 2019 for humans was used. Enriched terms with a top score and a p-value less than 0.05 were selected.

### Analyzing Gene Expression Profiles

Genes were given to GEPIA2 webserver to validate identified DEGs based on datasets in TCGA genomic database (43, 44). To draw boxplots, expression profiles were compared between tumor and normal samples in multiple colorectal adenocarcinoma (COAD) datasets. LogFC cutoff was set at 0.5 and p-value was set at 0.01. TPM normalized data were log2 transformed. To draw survival plots, “Overall Survival” option was selected and median was chosen to define the border of High and Low groups. 95% confidence interval was set for analysis. All COAD datasets with monthly expression values were selected in order to obtain survival results.

## Results

### Data Preprocessing in Test Datasets

**Figure 2:**
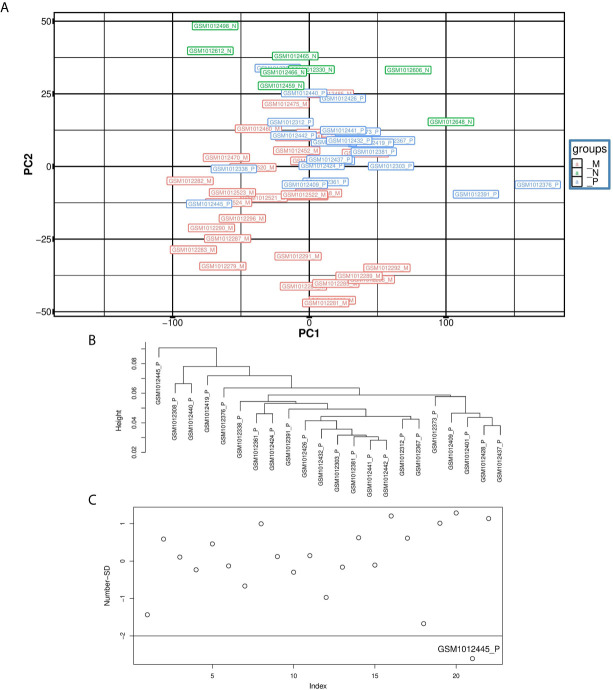
Illustration of outlier samples in the first dataset. **(A)** is the PCA plot, **(B)** is the dendrogram for the primary samples and **(C)** is the Number-SD plot for primary samples. GSM1012445 is an outlier sample in primary group as it has located in a distance from its group in the PCA plane. In addition, it has formed a unique cluster in the dendrogram and its Number-SD score is less than negative two.

Each dataset was imported into R separately. Outlier sample detection was conducted using PCA and hierarchical clustering approaches. **Figure 2A** illustrates the PCA plot for samples in Dataset1. The same plot was created for the second and third datasets. Some samples in the PCA plane lay at a distance from their group, particularly along the PC1 axis, so they were considered as the outliers. To be more specific, a hierarchical clustering method introduced by Oldham MC, et al. (45) was used. To compute the distances between samples, Pearson correlation analysis was performed between them, and coefficients were subtracted from one. **Figure 2B** depicts the dendrogram for normal samples. In **Figure 2C** normal samples were plotted based on their Number-SD scores. To get this number for each sample, the average of whole distances was subtracted from the distance average of each sample. Then, results of these subtractions were normalized by standard deviation of sample distance averages (45). Samples with Number-SD less than negative two usually fall apart from their cluster set in the PCA plane. Thus, they were regarded as the outliers in our analyses. Sixteen outlier samples in GSE41258 Test dataset and three outliers in Dataset3 were recognized. **Supplementary File 1** contains information about the groups of samples.

**Supplementary File 2** illustrates the average expression values for some housekeeping genes and DEGs between Primary and Metastatic samples. Common DEGs between lung-primary analysis and liver-primary analysis with absolute LogFC larger than one in GSE41258 datasets were illustrated in A. The same plot was made for the common DEGs in liver-primary analysis in the third dataset in B. Housekeeping genes were situated on the diagonal of the plots whilst DEGs were located above or under the diagonal. Hence, the preprocessed data were of sufficient quality for downstream analyses.

### Meta-Analysis and Identifying Differentially Expressed Genes

10891 unique DEGs with adjusted p-value < 0.05 and absolute log fold change > 0.5 were achieved from eight groups of DEGs yielded from eight independent analyses on three Test datasets. They included two analyses of liver metastasis versus normal, two analyses of liver metastasis versus primary, one analysis of lung metastasis versus normal, one analysis of lung metastasis versus primary, and two analyses of primary versus normal (**Table 1**). Liver metastasis contained metastatic colorectal samples taken out from liver; however, lung metastasis contained metastatic colorectal samples obtained from lung. In fact, this kind of dataset selection provided us with some DE genes that could significantly contribute to tumor progression within the primary site and towards liver and lung organs. Common DEGs between all metastasis vs normal analyses (Test datasets) were 155 genes. Common DEGs between all metastasis vs primary analyses (Test datasets) were 72 genes. Common DEGs between all primary vs normal analyses (Test datasets) were 239 genes. There were 334 unique DEGs between these three sets of analyses. Finally, from these 334 DEGs, 242 of them were identified to be in all Test and Validation analyses considered as the final DEGs. There were 12 final DEGs in primary versus normal analyses considered as the Core genes. All DE gene sets and their LogFC are presented in **Supplementary File 3**.

**Table 1:** The practical information for the Core network DE genes in Test dataset.

MvsN				BvsN		
DEGs	GSE41258		GSE9348_GSE10961	DEGs	GSE41258	GSE9348_GSE10961
	liver-normal (D1)	lung-normal (D2)	liver-normal (D3)		primary-normal (D1/2)	primary-normal (D3)
ETHE1	Down	Down	Down	SGK1	Down	Down
DARS	Up	Up	Up	EIF2S2	Up	Up
TMEM131	Down	Down	Down	TRIB3	Up	Up
TST	Down	Down	Down	DARS	Up	Up
LGALS4	Down	Down	Down	RFC3	Up	Up
TRIB3	Up	Up	Up	ETHE1	Down	Down
COL5A2	Up	Up	Up	TOP2A	Up	Up
COL4A1	Up	Up	Up	CKS2	Up	Up
PTP4A1	Down	Down	Down	SORD	Up	Up
COL1A2	Up	Up	Up	PSMA7	Up	Up
SORD	Up	Up	Up	GPX3	Down	Down
SQOR	Down	Down	Down	MAD2L1	Up	Up
OAS1	Down	Down	Down	SQOR	Down	Down
TRIM31	Down	Down	Down	TST	Down	Down
TWF1	Down	Down	Down	GINS1	Up	Up
**MvsP**				LGALS4	Down	Down
DEGs	GSE41258		GSE9348_GSE10961	PTP4A1	Down	Down
	liver-primary (D1)	lung-primary (D2)	liver-primary (D3)	PLAGL2	Up	Up
ETHE1	Down	Down	Down	COL1A2	Up	Up
ATF5	Up	Up	Up	CTSH	Up	Up
CDC6	Down	Down	Down	MT2A	Down	Down
TMPO	Down	Down	Down	COL5A2	Up	Up
P4HA1	Up	Up	Up			

“Up” means gene was upregulated, and “Down” means gene was downregulated. MvsN contains all the metastatic versus normal analyses, PvsN contains all the primary versus normal analyses and MvsP contains all the metastatic versus primary analyses. D stands for Dataset, so D1 means Dataset1. Some genes are present in more than one analysis.

### Undirected Protein-Protein Interaction Network

**Figure 3:**
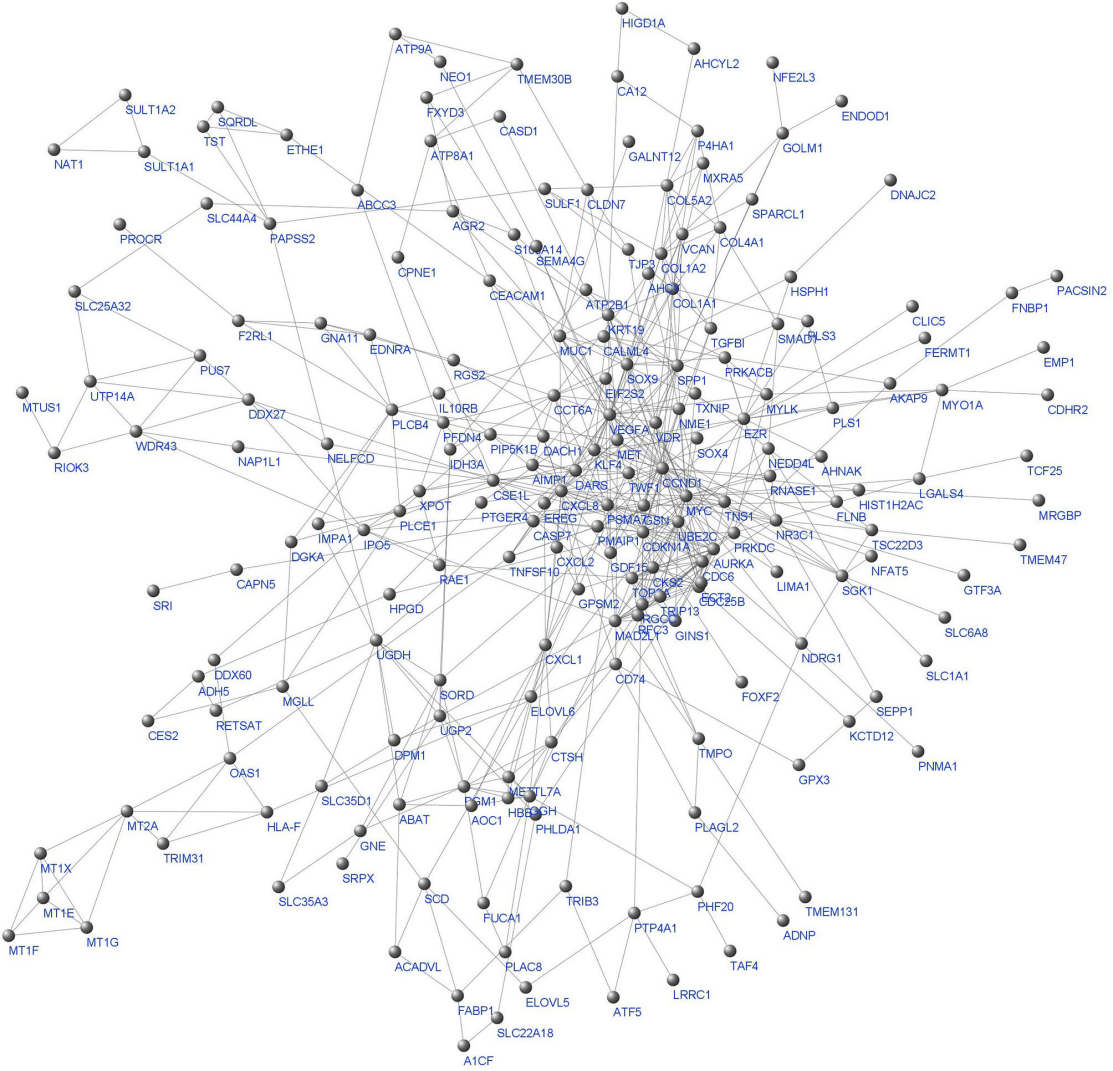
The whole network giant component. Labels are protein/gene symbols. This is a scale free network (46) which follows a power law distribution (most of the network nodes have a low degree while there are few nodes with high degree).

242 final DEGs were utilized to construct the Protein-Protein-Interaction (PPI) network. STRING database was employed to generate the Interactions based on seven sources of evidence, namely Neighborhood, Text mining, Experiments, Databases, Co-expression, Gene fusion, and Co-occurrence. STRING combined scores were used as the edge weights. The giant component of the weighted network with 205 nodes and 554 edges is depicted in **Figure 3**.

### Determination of Core Genes Neighborhood Through Shortest Path-Based Scoring System

**Figure 4:**
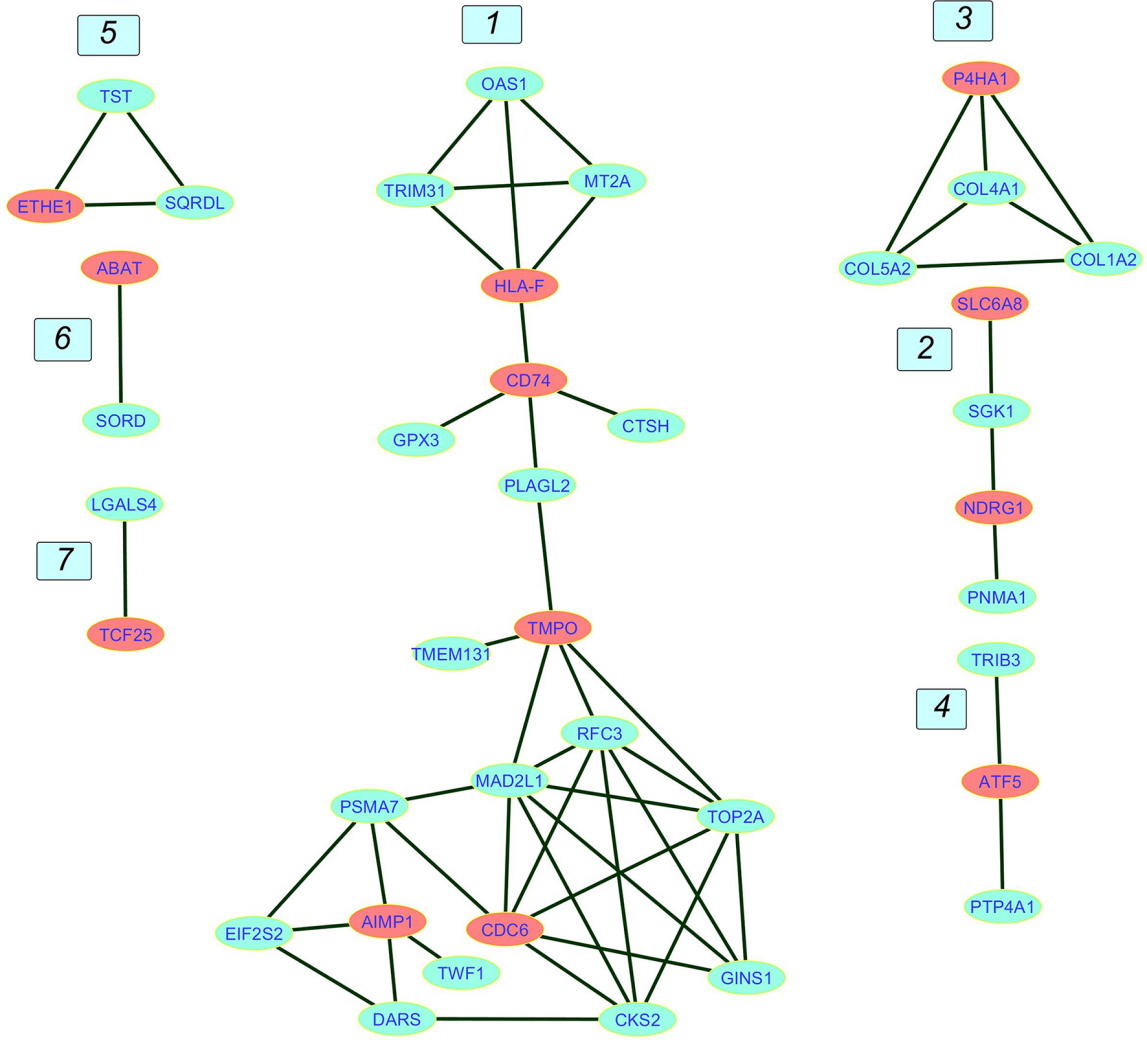
The Core network. The network contains seven components numbered from 1 to 7. Component 1 is the giant component. Core genes are in red and non-core genes are in blue.

In this step, interactions combined score computed from all sources of evidence in STRING database were converted into weights between nodes. These weights were used as the estimation of distances in the weighted adjacency matrix. Nodes with shorter distances from the Core genes were selected, and a smaller network was extracted from the main network. Computing the shortest path score for the non-core genes led to a network of 39 nodes comprising 12 Core nodes and 27 neighbors. This multi-component graph called Core network is illustrated in **Figure 4**.

**Figure 5:**
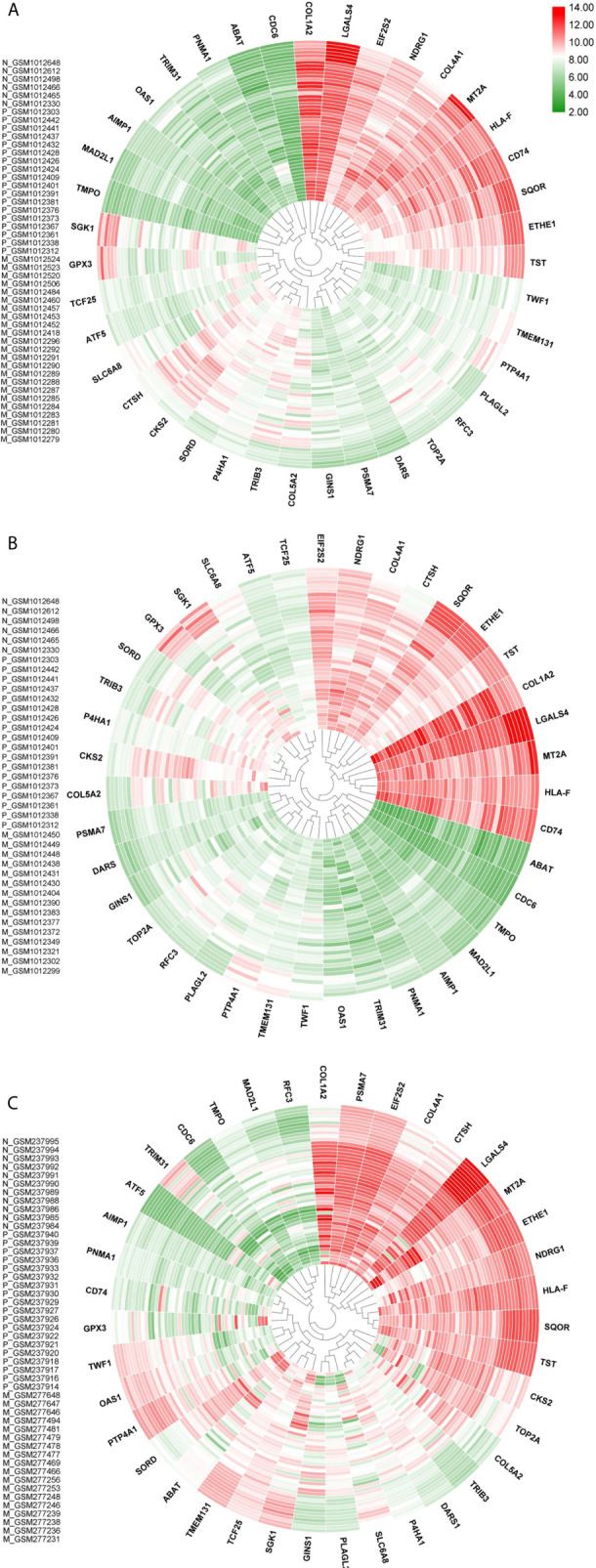
Core network genes heatmaps. **(A)** is dataset1, **(B)** in dataset2 and **(C)** is dataset3. Values were obtained from expression matrices, Log2 transformed and normalized by quantile method. Sample IDs are placed in the left-hand side of each plot. Normal samples start with N, Primary samples start with P and liver and lung metastatic samples start with M. Outer samples are normal, middle samples are primary and inner samples are metastatic. Genes were clustered together based on hierarchical clustering.

Majority of the nodes in Core network were selected for investigation based on the similarity of expression patterns in all datasets. Expression status of selected genes between any pairwise comparisons was depicted in **Table 1**. For the three Metastatic-Normal comparisons (MvsN), most of the nodes exhibited a similar expression pattern. The same was true for all Primary-Normal analyses (PvsN) and Metastatic-Primary comparisons (MvsP). Heatmaps were illustrated in **Figure 5** for all members of the Core network in three datasets. Clustering was performed by applying “Euclidean” distance and “Complete” method on gene expression values. Genes present in the top right corner of the three plots possessed high expression values in colon tissues. Moving from border to the center of plots, we go from Normal to primary and from primary to metastatic samples. Some genes exhibited a descending expression trend such as mitochondrial genes ETHE1, TST and SQOR. Few genes witnessed an ascending trend, such as collagen genes and SORD and P4HA1.

### Network Descriptive

The giant component diameter was eight containing TRIM31, HLA-F, CD74, PLAGL2, TMPO, MAD2L1 PSMA7, AIMP1, and TWF1. Transitivity was around 60%, edge density was about 18%, and the mean distance was 3.48. Two important centralities, Degree and Betweenness, along with other centralities and the average distances for giant component nodes, are provided in **Supplementary File 4**. MAD2L1 had the highest Degree and a relatively high betweenness. TMPO had the highest Betweenness and a pretty high degree. Similar to TMPO, its direct neighbor, PLAGL2 had a relatively high Betweenness. This gene has linked the upper and lower parts of the PPI giant component together.

### Processing Validation Datasets

Core network nodes were identified in the seven Validation datasets. They were presented in **Table 2**. Most of the DEGs illustrated similar results in both **Tables 1**, **2**, which proves the accuracy of obtained DEGs from Test datasets. Expression of genes that were totally homogeneous in each of MvsN or MvsP or PvsN analyses are presented in green, and the ones that differed only in one analysis are shown in yellow. Expression of Genes that were different in more than one dataset are in white. Absolute LogFCs less than 0.2 were not reported in **Table 2**. Expression analysis of all Validation datasets are presented in **Supplementary File 3**.

**Table 2:** Illustration of Core network DEGs in Validation datasets.

MvsN							PvsN					
DEGs	GSE40367	GSE50760	GSE41258		GSE144259	GSE89393	DEGs	TCGA	GSE50760	GSE41258	GSE144259	GSE89393
	liver-normal	liver-normal	liver-normal	lung-normal	liver-normal	liver-normal		primary-normal				
ETHE1	Down	Down	Down	Down	Down	Down	SGK1	Down	Down	Down	Down	Down
DARS	Up	Up	Up	Up	Up	Up	EIF2S2	Up	Up	Up	Up	Up
TMEM131	Down	Down	Down	Down	Down	Down	TRIB3	Up	Up	Up	Up	Up
TST	Down	Down	Down	Down	Down	Down	DARS	Up	Up	Up	Up	Up
LGALS4	Down	Down	Down	Down	Down	Down	RFC3	Up	Up	Up	Up	Up
TRIB3	Up	Up	Up	Up	Up	Up	ETHE1	Down	Down	Down	Down	Down
COL5A2	Down	Up	Up	Up	Up	Down	TOP2A	Up	Up	Up	Up	Up
COL4A1	Down	Up	Up	Up	Up	Down	CKS2	Up	Up	Up	Up	Up
PTP4A1	Down	Down	Down	Down	Down	Down	SORD	Up	Up	Up	Up	Up
COL1A2	Up	Up	Up	Up	Up	Up	PSMA7	Up	Up	Up	Up	Up
SORD	Up	Up	Up	Up	Up	Up	GPX3	Down	Down	Down	Down	Down
SQOR	Down	Down	Down	Down	Down	Down	MAD2L1	Up	Up	Up	Up	Up
OAS1	Down	Down	…	Down	Down	Down	SQOR	Down	Down	Down	Down	Down
TRIM31	Down	Down	Down	Down	Down	Down	TST	Down	Down	Down	Down	Down
TWF1	Down	Down	…	Down	…	Down	GINS1	Up	Up	Up	Up	Up
**MvsP**							LGALS4	…	Down	Down	Down	Down
DEGs	GSE40367	GSE50760	GSE41258		GSE144259	GSE89393	PTP4A1	Up	Down	Down	Down	Down
	liver-primary	liver-primary	liver-primary	lung-primary	liver-primary	liver-primary	PLAGL2	…	Up	Up	Up	Up
ETHE1	Down	Down	…	Down	Down	Down	COL1A2	Down	Up	Up	Up	Up
ATF5	…	Up	Up	…	Up	…	CTSH	…	Up	Up	Up	Up
CDC6	Down	Up	Down	Down	Down	Down	MT2A	Down	Down	Down	Down	Down
TMPO	Down	…	…	Down	Down	Down	COL5A2	Down	Up	Up	Up	Up
P4HA1	Up	Up	Up	Up	Up	Up						

Up means gene was upregulated and Down means gene was Downregulated. MvsN contains all the metastatic versus normal analyses, PvsN contains all the primary versus normal analyses and MvsP contains all the metastatic versus primary analyses. Some genes are present in more than one analysis. The expression status for the genes in green rows are similar in all datasets regardless of empty cells. In the yellow rows only one dataset is different from the others and in the white rows genes exhibited a heterogeneous expression status in different datasets. Green rows illustrate a similar expression in all datasets.

### Over-Representation Analysis

**Figure 6:**
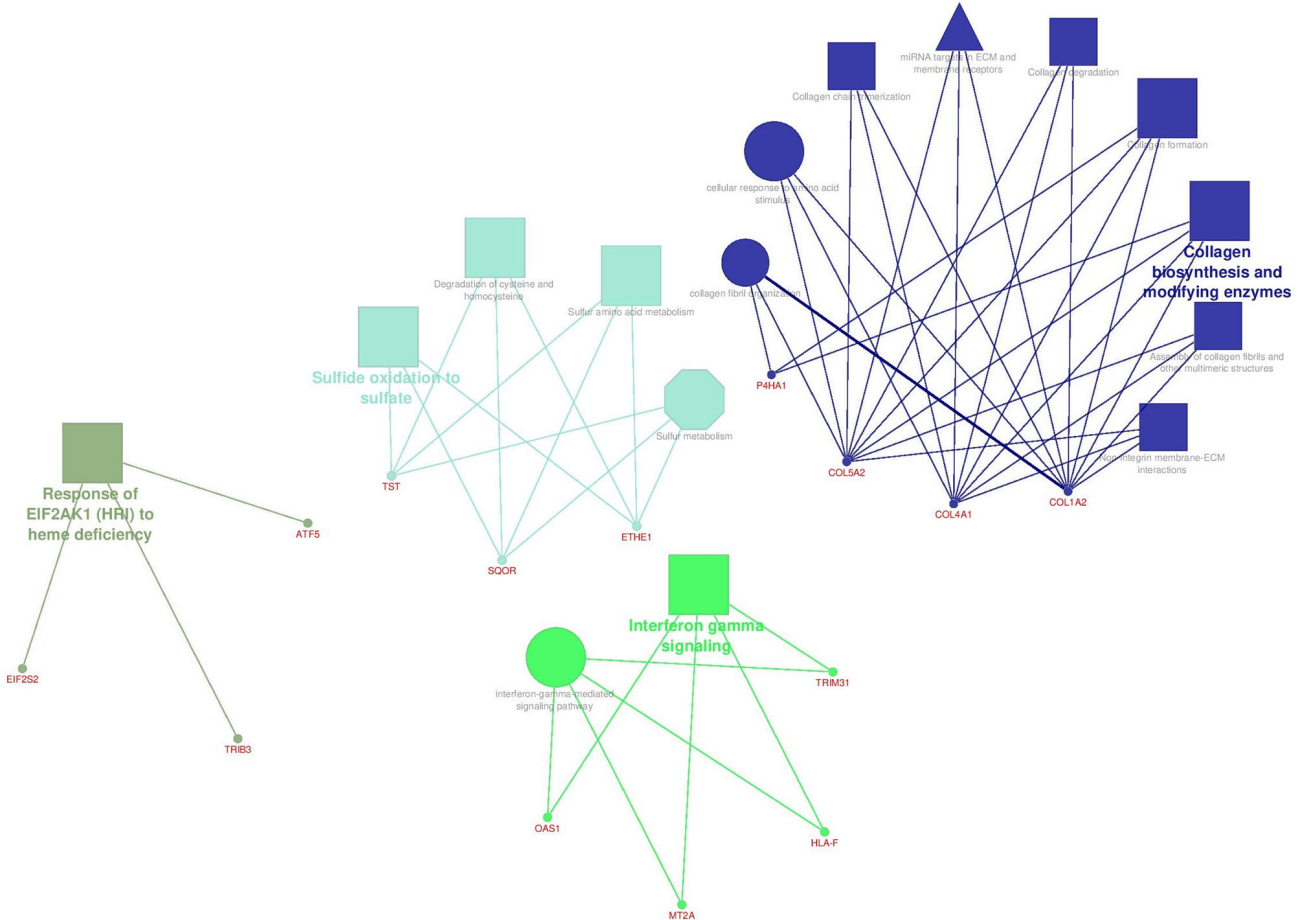
The enrichment results for the Core network genes. Terms in the shape of octagons are from KEGG, Triangular terms are from WikiPathways, rectangular expressions are from Reactome and circular terms are from GO database. Size of the terms present their significance.

**Figure 6** illustrates the enrichment results for the Core network genes using ClueGO software. Three signaling databases called KEGG, Reactome, and WikiPathways were used for the pathway enrichment. Biological Function terms were enriched from GO database. Genes and terms associated with a specific cellular mechanism formed distinct components. Different pathway terms related to polymerization and degradation of collagens in extra cellular matrix (ECM) have emerged in blue, which formed a distinct component (component 3) in the Core network. In a tumor environment, different concentrations of collagen fibers are regularly secreted, degraded, and aligned together to make ECM stiffness suitable for cellular migration (47, 48). Genes that were enriched for sulfide oxidation terms formed a distinct component in the Core network as well. Genes in green are engaged in interferon-gamma signaling that has dual roles in cancer. On the one hand, INF-γ has anti-proliferative functions by employing different mechanisms such as induction of p21 (49), induction of autophagy (50), regulation of EGFR/Erk1/2 and Wnt/β-catenin signalings (51), and so on. On the other hand, it enhances the outgrowth of tumor cells with invasive properties depending on cellular and microenvironmental context (52, 53).

In the enrichment analysis with “Enrichr” online tool, Gastric Cancer Network2 was of the lowest p-value containing TOP2A and RFC3 genes involved in DNA replication process. Involvement of the same genes in retinoblastoma cancer (WP2446) proposes the potential importance of these genes in different cancers. Top2A was involved in Gastric Cancer Network1 as well. All the enrichment results yielded from “Enrichr” are presented in **Supplementary File 5**.

### Expression Profiling and Survival Analysis of TCGA Gene Expression Profiles

**Figure 7:**
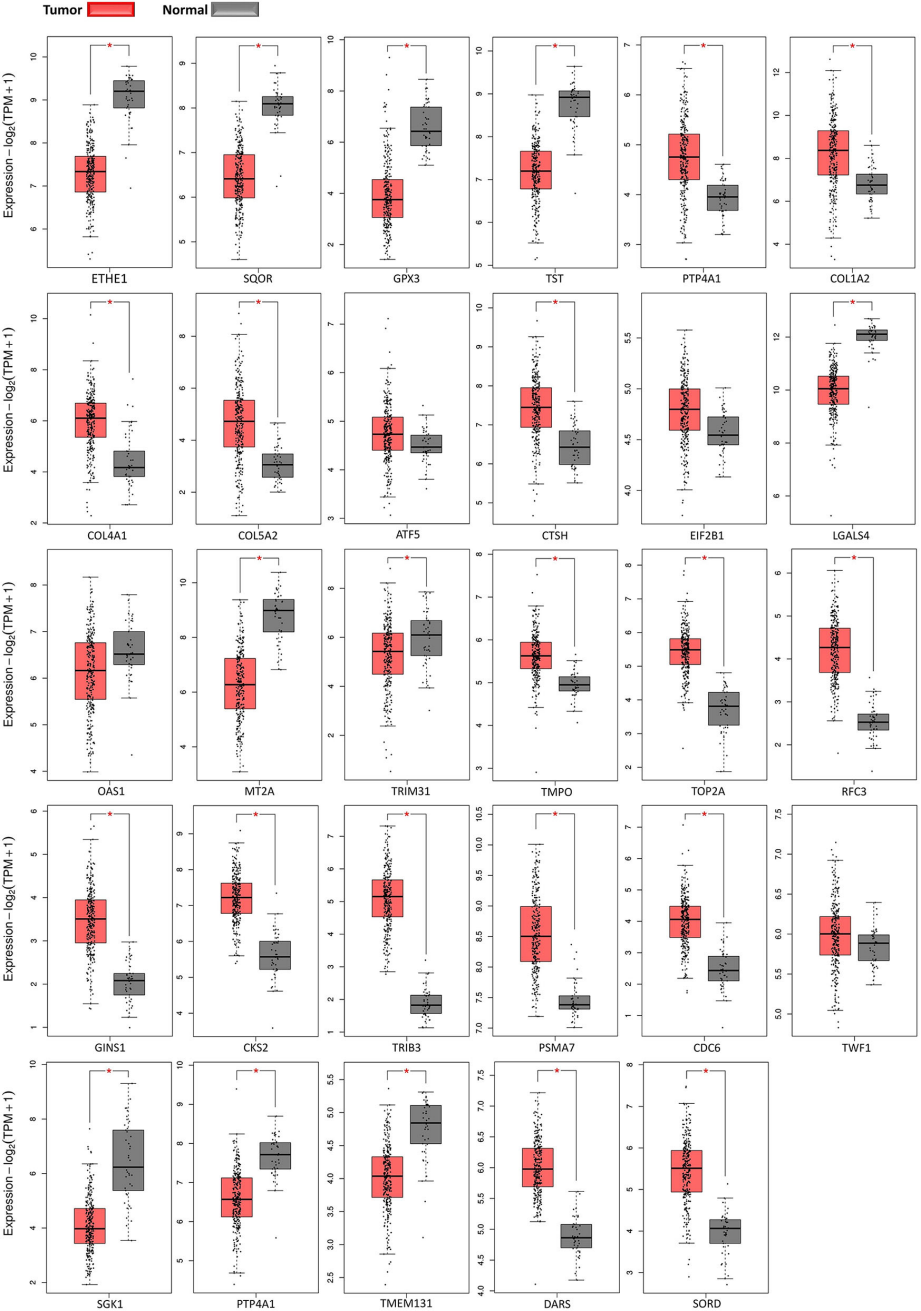
Gene expression profiles boxplots. Red boxes present normalized expression values for tumor adenocarcinoma samples and gray boxes present values for normal colon samples.

Expression of DEGs in **Tables 1**, **2** were further supported by boxplots and survival plots using GEPIA2 web server. Expression profiles were attained from 275 colorectal adenocarcinoma and 41 normal colon RNA-seq samples in TCGA database to create boxplots for each gene in **Figure 7**. Except for TWF1, all plots were in agreement with our results. In other words, if a gene was upregulated in our analysis between cancer and normal groups, the expression median for that gene in tumor samples was larger than normal samples in boxplots and vice versa. Even boxplots for expression of some genes that were later shown to be contradictory to other studies, were in favor of our findings. They were MT2A, TRIM31, CDC6, SGK1, and PTP4A1 genes.

**Figure 8:**
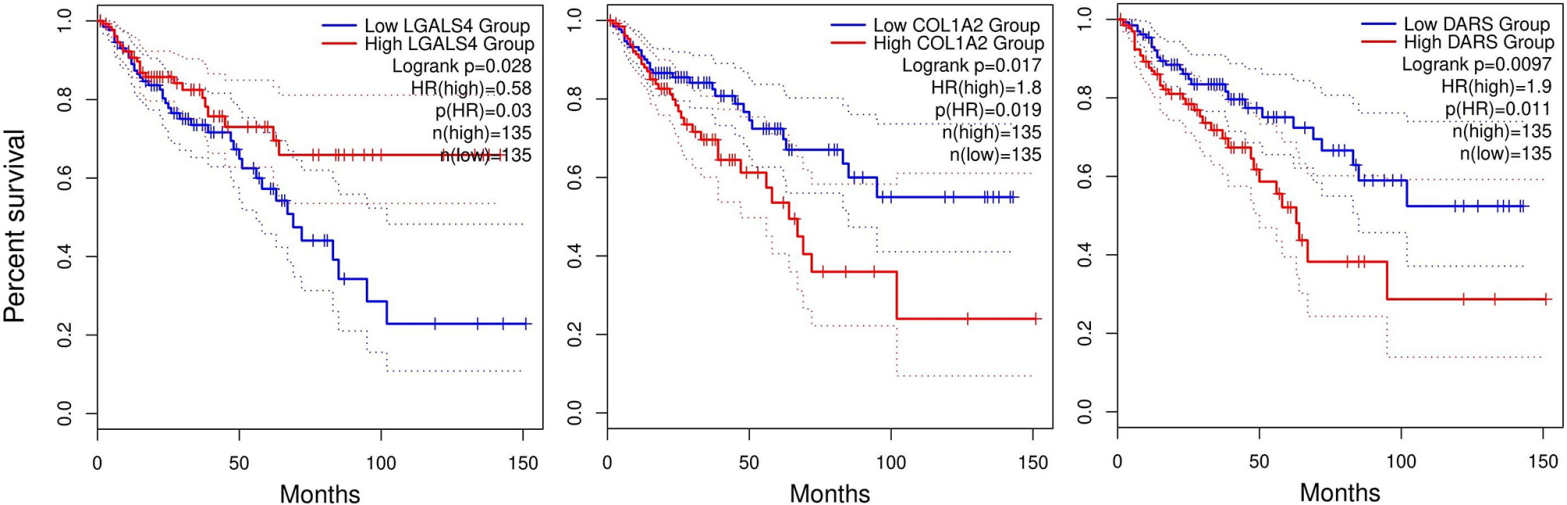
Survival plots. Plots present the monthly survival rate of patients having high expression, red line, or low expression, blue line, of a specific gene. Patients having high expression of LGALS4 had higher survival rates compared to patients having low expression of LGALS4. Contrary, patients having low expression of COL1A2 and DARS genes had higher survival rate.

Survival plots were also created for DEGs in **Tables 1**, **2** in different months using TCGA database. Only three genes had a significant p-value larger than 0.05 illustrated in **Figure 8**, and rest of survival plots were presented in **Supplementary File 6**. Low expression of LGALS4 is associated with poor survival rate, while high expression of COL1A2 and the new reported gene DARS is linked to poor survival rate in colorectal cancer patients. In our study, LGALS4 were downregulated in MvsN and PvsN analyses of all Test and Validation datasets while DARS and COL1A2 were upregulated in majority of MvsN and PvsN analyses. Although other survival tests were non-significant, majority of them were in agreement with our expression results.

## Discussion

The Core network giant component is composed of an up and a down part attached *via* PLAGL2 transcription factor (TF). The lower part is engaged mainly in cell cycle and DNA replication. Components 2 and 3 contain genes involved in ECM remodeling, component 4 is composed of genes involved in transcription inhibition, and Component 5 is composed of mitochondrial genes playing essential roles in controlling cellular redox homeostasis. Here we discussed most of the genes in the Core network exhibiting more similar expression patterns which were present in **Tables 1**, **2**.

PLAGL2 is considered an oncogene in different cancers. It binds to and prevents Pirh2 proteasomal degradation, which in turn Pirh2 promotes proteasomal degradation of P53 protein (54). In glioblastoma, PLAGL2 suppresses neural stem cell differentiation by regulating Wnt/β-catenin signaling (55). Besides, PLAGL2 regulates actin cytoskeleton and cell migration through promoting actin stress fibers and focal adhesion (56). Results of PvsN analysis manifests that this gene is induced in primary tumors in colon cancer. In addition, this gene had a high betweenness centrality in the giant component (S4). Since this gene connected the two parts of the giant component, it would be a pertinent target for disturbing colon cancer network. Its induction in CRC was supported by the majority of Validation datasets in **Table 2**.

TRIM31 (a ubiquitin ligase) was downregulated in MvsN in all Test and Validation datasets. However, there are contradictory results in different studies where it was shown to be reduced in lung cancer cells (57) and stepped up in gastric (58) and colorectal cancer (59). Therefore, its downregulation in nine analyses in **Tables 1**, **2** needs to be further explored. MT2A gene is an antioxidant that protects cells against hydroxyl radicals and plays an important role in detoxifying heavy metals (60, 61). Expression inhibition of this gene results in proliferation inhibition of CRC cells (62), and its silencing promotes the effects of chemotherapy drugs on primary osteosarcoma tumors (63). However, MT2A gene expression was downregulated in PvsN analyses supported by the results in **Table 2**. Likewise, it is downregulated in pancreatic cancer as well (64). Therefore, this downregulation in primary CRC tumors has to go under more investigation. OAS1 is a protein induced by interferons that synthesizes the oligomers of adenosine from ATP. These oligomers bind to RNase L to regulate cell growth, differentiation, and apoptosis (65). Its expression is downregulated in breast ductal carcinoma and prostate cancer (PCa) at both mRNA and protein levels. In addition*, *OAS1 expression is negatively correlated with the progression of these cancers (65). The given information supports the downregulation of this gene in our analysis supported by Validation datasets. Consequently, expression induction of this gene might help prevent both tumor growth and cell differentiation. The mentioned three genes, TRIM31, MT2A and OAS1, were enriched for IFN-γ and all were downregulated. Although there are contradictory results in different papers, these downregulations at mRNA level would help tumor cells to defeat the anti-cancer properties of interferon gamma signaling.

CTSH gene is a lysosomal cysteine protease upregulated in PvsN. This protease plays an important role in cytoskeletal protein Talin maturation. Talin promotes integrin activation and focal adhesion formation leading to cell migration (66). Validation datasets more verified upregulation of this gene in CRC. As a result, suppression of CTSH expression could be a choice of metastasis inhibition. Glutathione peroxidase 3 (GPX3) is an important antioxidant enzyme that protects cells against Reactive Oxygen Species (ROS), downregulated in many cancers. For instance, its expression is suppressed in human colon carcinoma Caco2 cell lines, resulting in augmented ROS production (67). It reduces H_2_O_2_ and lipid peroxides to water and lipid alcohols, respectively, and in turn, oxidizes glutathione to glutathione disulfide (68). Downregulation of GPX3 happened in PvsN analyses, leading to ascending of H_2_O_2_ level, which is positively correlated with tumor progression (69). Its downregulation was further supported by all datasets in **Table 2**. As a result, induction of GPX gene families would be a therapeutic approach.

TMPO gene had the greatest Betweenness centrality illustrating a reduced expression trend in MvsP analyses supported by Validation datasets. This gene produces different protein isoforms *via* alternative splicing (70, 71). The proteins are located in the nucleus of the cells, which help form nuclear lamina and maintain nucleus membrane structure (72). TMPO prevents the depolymerization of nuclear laminas and excessive activation of the mitosis pathway. Therefore, its downregulation would prevent an excessive mitotic cycle.

TMEM131 is a transmembrane protein that was downregulated in MvsN analyses in all datasets in **Tables 1**, **2**. No documentation was found to connect this gene to a specific cancer. Therefore, this gene might be biomarker of CRC. Furthermore, Enrichment analysis using “Enrichr” online tool showed that this gene was also involved in interferon-gamma signaling (S5). A recent study has discovered that amino termini of human TMEM131 recruit monomers of collagens for assembly. Carboxy termini of this gene guide collagen cargo machinery from Endoplasmic Reticulum towards Golgi apparatus, contributing to collagen maturation and secretion. Moreover, TMEM131 deficiency diminishes collagen production, maturation, and secretion in Caenorhabditis Elegans (73). This gene is also important for ER processing of cuticle collagen cargos and apical ECM (aECM) formation in Drosophila melanogaster. These findings highlight the conserved role of this gene in collogen biosynthesis (74). The methylation rate of this gene is reduced in T-Cells and peripheral blood cells in Down syndrome patients (Trisomy 21). This gene also marks lymphocyte precursor cells for lineage specification (75).

TOP2A gene was upregulated in PvsN analyses entirely endorsed by the validation results. In breast cancer (BC) HER‐2 and TOP2A are the molecular targets for several anticancer medicines that are bolstered together (76). Moreover, Copy Number Variations (CNVs) in TOP2A gene have been identified as biomarkers of colorectal cancer (77). This enzyme controls DNA topological structure, and its upregulation is a hallmark of aberrant cell growth in tumors (78). TOP2A mRNA expression is an independent prognostic factor in patients with (Estrogen Receptor) ER-positive breast cancer and could be useful in the assessment of breast cancer risk (79). Therefore, in addition to being a possible target for CRC therapy, this gene could be either a possible prognostic or diagnostic marker of CRC.

Replication Factor C subunit 3 (RFC3) plays a role in DNA replication, DNA damage repair, and cell cycle checkpoint control. Hepatocellular carcinoma (HCC) and cell proliferation of ovarian tumors are suppressed by shRNA-mediated silencing of RFC3 gene (80, 81). This gene was upregulated in PvsN analyses and is upregulated in Triple-negative breast cancer (TNBC) as well (82). Validation datasets more supported its upregulation. Since expression inhibition of this gene at both mRNA and protein levels suppresses the migratory and invasive ability of MCF-7 cell lines (83), this gene would be a therapeutic target for colorectal cancer treatment. Moreover, TOP2A and RFC3 were shown to be engaged in the Gastric Cancer Network2 pathway in the enrichment analysis by “Enrichr” (S5), indicating the importance of these two genes in cancer progression.

Mitotic Arrest Deficient 2 Like1 (MAD2L1) is a mitotic spindle assembly checkpoint molecule upregulated in PvsN in both Test and Validation analyses. It is responsible for preventing anaphase initiation until precise and complete metaphase alignment of all chromosomes takes place. An increase in the level of MAD2L1 transcripts is detected in a large number of samples with ductal breast carcinoma (84). Its upregulation in our analysis would provide evidence that cancerous cells were dealing with mitotic deficiencies. The GINS complex is a DNA replication machinery component in the eukaryotes and is an essential tool for initiating and progressing DNA replication forks (85). GINS1 (PSF1) mRNA level is positively correlated with tumor size in CRC patients and is a prognostic marker of CRC (86). This gene has been recently introduced as a targeted oncogenic agent for inhibition of synovial sarcoma (87). It was totally upregulated in PvsN analyses in **Tables 1**, **2**. Therefore, its expression inhibition would be a potential target for inhibition of tumor growth by disturbing DNA replication machinery.

CDC6, one of the Core genes, plays a critical role in regulation of the eukaryotic DNA replication onset, and its downregulation has been demonstrated in prostate cancer (88). It is a regulator of cell cycle in S phase, and its expression is regulated by E2F Transcription factor and androgen receptors (AR) in PCa cells (89). Transfection of CDC6 siRNA leads to not only decreased level of ovarian cancer cell proliferation but also increased apoptosis rates (90). Cdc6 and Cdt1 are highly expressed in aggressive BC and therefore is considered a potent therapeutic target in BC patients (91). Results for this gene in MvsP analyses were contradictory to the BC results, but it is similar to prostate cancer. The majority of Validation datasets depicted downregulation of this gene in CRC. No study directly measured the expression level of this gene in CRC samples; therefore, it is worth investigating to see whether it could be a CRC biomarker or a curative target.

CKS2 protein interacts with the catalytic subunit of the cyclin-dependent kinases, and its downregulation contributes to suppression of p-Akt and p-mTOR. Therefore, one of CSK2 oncogenic roles is played by Akt/mTOR oncogenic pathway (92). CKS2 is expressed at a high level in CRC tissues, and it has revealed that increased CKS2 expression is highly correlated with enhanced metastatic stage (93). Importantly, CKS2 is considered a potential biomarker and therapeutic target for the BC treatment due to the fact that its inhibition suppresses cell proliferation and invasion *in vitro* and *in vivo* (94). In the PvN analyses, this gene was upregulated, which would be a therapeutic target for CRC treatment because validation results completely supported this upregulation.

PSMA7 gene encodes a protein that is one of the essential subunits of 20S proteasome complex (95). Overexpression of PSMA7 both at the mRNA and protein levels has been reported in gastric cancer (96). Depletion of PSMA7 by shRNA-transfected RKO CRC cell lines mediates inhibition of cell growth and migration. Consequently, inhibition of PSMA7 could be a beneficial therapeutic strategy for colorectal cancer patients (97). This gene was upregulated in PvsN analyses in test and Validation datasets.

DARS encodes the cytosolic aspartyl-tRNA synthetase found to be upregulated in MvsN and PvsN analyses in all Test and Validation datasets (a total of 16 analyses). This gene encodes a member of a multi-enzyme complex that its role has been proved in mediating attachment of amino acids to their cognate tRNAs. Some studies have reported that DARS-AS1 gene (encoding a long noncoding RNA) act as an oncogene (98) and is positively associated with the pathological stages in thyroid and ovarian cancer by targeting mir-129 and mir-532-3p, respectively (99, 100). Moreover, this gene is directly upregulated by HIF1 gene, which stabilizes RBM39 protein in Myeloma (101). Mutations in this gene have been previously reported in neuroinflammatory diseases and Leukodystrophies (102, 103). However, there is not enough evidence in the literature that associates DARS1 (DARS) gene to different cancers. Moreover, patients having a lower expression of this gene have a higher survival rate in **Figure 8**.

EIF-2 consists of alpha, beta, and gamma subunits. EIF2B or EIF2S2 acts in the early steps of protein synthesis. GTP-bound EIF-2 transfers Met-tRNAi to the 40S ribosomal subunit to start protein synthesis. The hydrolysis of GTP to GDP takes place at the end of the initiation process that leads to release of the inactive eIF2·GDP from ribosome. Exchange of GDP for GTP is performed by beta subunit so that active EIF-2 is ready for another round of initiation (104). In one study, EIF2B was proposed as a potential therapeutic target in lung cancer (76). Moreover, elimination of natural killer cell cytotoxicity *via* promoted expression of natural killer (NK) cell ligands is done by pSer535-eIF2B following the expression of pSer9-GSK-3β (inactive GSK3β) and generation of ROS, which promotes breast cancer growth and metastasis (105). Since Tyr216-GSK-3β (Active GSK3β) has inhibitory effects on the EMT process by interfering with TNF-alpha signaling (106), induction of active GSK-3β together with suppression of EIF2B would be a therapeutic approach to prevent EMT (107). EIF2B stepped up in PvsN analyses which was supported by validation results.

TWF1 gene encodes Twinfilin, an actin monomer-binding protein that promotes EMT in pancreatic cancer tissues (108). TWF1 siRNA dramatically inhibits F-actin organization and focal adhesions formation, promoting the mesenchymal-to-epithelial transition (MET) in MDA-MB-231 cell lines. Besides, The responsiveness of these cell lines to anti-cancer drugs such as doxorubicin and paclitaxel is augmented by siRNA inhibition of TWF1 expression (109). Furthermore, expression levels of EMT markers, VIM and SNAI2, are reduced due to miR-30c action on TWF1 mRNA (109). However, in MvsN analyses, this gene witnessed a decreased expression in both Test and Validation datasets. As a result, Its upregulation in CRC has to be further explored.

SGK1, a member of component 2, and AKT are two families of AGC protein superfamily. SGK1 is a serine/threonine kinase that activates particular potassium, sodium, and chloride channels (110). SGK1 is a downstream effector of PI3K, which runs pathways independent of pathways shared with AKT. The two kinases are phosphorylated and activated by PDK1 and mTORC2 complex (111, 112). In general, PI3K-dependent survival signals can be mediated by either Akt or SGK1 that inactivates the pro-apoptotic proteins Bad and FKHRL1 (113). A study on A498 kidney cancer cells found that survival signals promoted by IL-2 are mediated by SGK1 activation (114). Moreover, the promoter of SGK1 is under tight control of the p53 protein (115). SGK1 has been shown to mediate cell survival and drug resistance to platinoid and taxane compounds in breast cancer patients (116). On the contrary, this gene was totally downregulated in PvsN analyses in all Validation and Test datasets. These overall downregulations might be specific to CRC, so it could be a diagnostic hallmark of CRC and should go under more interrogation.

Component 3 contains collagen (COL1A2, COL5A2, and COL4A1) and P4HA1 (a collagen hydroxylase) genes interconnected in the process of ECM remodeling based on the enrichment results. All members witnessed an ascending trend in expression from normal samples to metastatic samples in **Figure 5** panels. In Test datasets, collagen genes presented an upregulation trend in MvsN and PvsN analyses, while their expression followed a mixed trend in Validation datasets. P4HA1 one of the Core genes upregulated in MvsP in all Test and Validation datasets. Expression of COL1A2 followed a homogeneous upregulating trend in both Test and Validation datasets which is a marker of EMT (117). P4HA1 is engaged in breast and pancreatic metastasis (118, 119). Under hypoxic tumor conditions, HIF-1 induces expression of genes that encodes collagen prolyl (P4HA1 and P4HA2) and lysyl (PLOD2) hydroxylases. P4HA1 and P4HA2 are required for collagen deposition, whereas PLOD2 is required for ECM stiffening and collagen fiber alignment (120). These changes in ECM triggered by HIF-1 are necessary for motility and invasion because, in focal adhesion junctions, actin cytoskeleton is connected to ECM through attachment of integrins to collagens (121). Besides, there is a positive feedback between P4HA1 and HIF-1 in modulation of ECM. As a result, targeting P4HA1 and P4HA2 expressions would inhibit the progression of cell migration *via* HIF1-Collagen pathway.

PTP4A1 a member of component 4, is a protein phosphatase engaged in p21-activated kinase (PAK) signaling pathway. Inhibition of PTP4A1 gene in MDA-MB-231 breast cancer cell lines by an increase in miR-944 expression impairs cell invasion (122). However, this gene was downregulated in MvsN and PvsN in all Test datasets and most Validation datasets. This downregulation would be a biomarker for CRC, and its molecular role in CRC needs to be interrogated. BCL-2 is a target of ATF5, one of the Core genes (123). ATF5 was upregulated in MvsP analyses in Test and Validation datasets. There are pieces of evidence that link the role of ATF5 in mitochondrial dysfunction in cancer progression (124). In malignant glioma, metastatic cells take advantage of survival signals triggered by ATF5 gene, which is essential to ignore anchorage-dependent and niche-dependent cell death signals (125). Thus, expression inhibition of ATF5 would hinder the survival signals in CRC cells. TRIB3 is a prognosis hallmark of colorectal cancer, activated under hypoxic conditions (126). TRIB3 silencing suppresses VEGF−A expression in gastric cancer cells inhibiting endothelial cell migration and vessel formation. This gene was upregulated in MvsN analyses in all Test and Validation datasets. Therefore, it would be a promising target for anti−angiogenic therapy (127).

Genes in component 5 are mitochondrial which their role in cancer progression has not been sufficiently investigated so far. All three genes were downregulated in our analysis in both Validation and Test datasets. They also exhibited a reducing trend from normal to primary and from primary to metastatic in **Figure 5** panels. These genes are highly expressed in normal colon tissue compared to other tissues due to the presence of anaerobic bacteria in the digestive tract (128). These findings are supported by the RNA-seq expression information in the Gene database of NCBI (129). ETHE1 (persulfide dioxygenase) and SQOR are antioxidants that convert hydrogen sulfide (H2S) to persulfide, then to sulfite. Hence, they protect cells against toxic concentrations of sulfide. ETHE1 gene was downregulated in the three analyses while SQOR was downregulated in MvsN and PvsN analyses. All these expressions were totally verified by the Validation datasets. Their downregulation is essential for cancer cells proliferation and survival. Under the hypoxic environment of CRC tumor, sulfide is a supplementary tool that provides electron for mitochondrial electron transport chain (ETC) to generate ATP (130). These mechanisms, along with Warburg effect help tumor cells to survive from the hypoxic environment. As a result, helping expression induction or activation of ETHE1 and SQOR proteins will increase sulfide scavenging and this would hinder CRC tumor growth. TST thiosulfate sulfurtransferase encodes a protein that is localized to the mitochondria and catalyzes the conversion of thiosulfate and cyanide to thiocyanate and sulfite respectively. Therefore, like the previous two mitochondrial enzymes, it acts in Hydrogen sulfide metabolism (131).

SORD (Sorbitol dehydrogenase) is another element of component 6 upregulated in MvsN, and PvsN analyses. Little is known about the connection between SORD and cancer. This enzyme hydrogenates Fructose to Sorbitol in Fructose catabolism pathway. Subsequently, sorbitol is dehydrogenated to glucose *via* AKR1B1 enzyme providing fuel for cells (132). In addition, excess glucose promotes EMT through autocrine TGFβ stimulation (133). Expression suppression of either enzyme reduces EMT in human lung cancer cells and EMT-driven colon cancer mouse model (133). Two studies demonstrated that SORD is an androgen-regulated gene in prostate cancer (134, 135). siRNA inhibition of this gene leads to proliferation and migration inhibition of A549 lung cancer cells (136). Since SORD exhibited an ascending trend in all Validation and Test datasets in **Figure 5**, it might be a potential target and biomarker to prevent EMT and cell growth in CRC. LGALS4 is implicated in regulating cell-cell and cell-matrix interactions, so its induction might have positive curative impacts on CRC cells. This gene is primarily expressed in small intestine, colon, and rectum, which is suppressed in CRC (137). It was downregulated in MvsN and PvsN analyses in Validation and Test datasets. It is also a blood marker of CRC (138).

In summary, we illustrated some therapeutic targets and biomarkers for CRC. A combination of these targets would beneficially disturb progression of colorectal cancer. Generally, the discovered antioxidants were downregulated in different stages of CRC, namely ETHE1, SQOR, TST, and GPX3. We proposed that these downregulations under hypoxic conditions would help cancer cells to produce more energy for cell proliferation. In addition, the hypoxic environment alters ECM suitable for cell migration by induction of P4HA1 gene through HIF-1 signaling pathway and induction of COL1A2. Boxplots (expression profiling) in **Figure 7** supported our results for all these genes. In addition, survival plot in **Figure 8** demonstrated that there is a higher death probability for CRC patients expressing a high level of COL1A2 than patients having a low level of this gene. Consequently, colorectal cancer cells would take advantages of explained mechanisms along with Warburg effect to not only survive from the hypoxic environment of tumors but also proliferate faster and migrate better. Therefore, induction of mentioned antioxidants and suppression of P4HA1 and COL1A2 genes would be a choice of CRC treatment.

Induction of active GSK-3β together with suppression of EIF2B would prevent EMT in CRC. Induction of OAS1 to increase the anti-cancer effects of interferon gamma, suppression of CTSH to hinder formation of focal adhesions, expression inhibition of ATF5 gene to make cancer cells sensitive to anchorage-dependent death signals, and induction of LGALS4 gene (supported by survival analysis) to recover cell-cell junctions would be the combination of genetic targets that prevent EMT and cell migration. In addition, expression inhibition of TMPO, TOP2A, RFC3, GINS1, and CKS2 genes could prevent tumor growth and TRIB3 expression suppression would be a favorable target for anti−angiogenic therapy. PSMA7 gene was a previously reported target for CRC treatment that was also found in our study. Results for expression of all these genes were supported by expression profiling.

MT2A and TRIM31 which were engaged in IFN-γ signaling, CDC6, SGK1 and PTP4A1 genes, presented a homogeneous expression pattern in both test and Validation datasets, although our results were contradictory to other studies in different cancers. Nevertheless, we used 10 different datasets from different technologies to ensure the accuracy of the results. Besides, expression profiling supported expression of these genes. However, they have to be further interrogated in colorectal cancer progression.

TMEM131 and DARS genes had specific uniform expression trends as analyses went from normal to metastatic. DARS expression inhibition would increase the survival rate in CRC patients based on **Figure 8**. Therefore, this gene might be a CRC prognostic marker or a curative target. Downregulation of TMEM131 might be associated with the amount of collagen secretion in ECM to make the environment suitable for migration (Stiffness of ECM). SORD is a poorly studied gene in cancer that its expression reduction might prevent cell proliferation and EMT in CRC. The relation of these three genes to colorectal cancer progression has been reported for the first time in this study. More investigation is required to find their molecular mechanism causing colorectal cancer promotion.

## Data Availability Statement

The datasets presented in this study can be found in online repositories. The names of the repository/repositories and accession number(s) can be found in the article/**Supplementary Material**.

## Author Contributions

MP: Conceptualization, Data Curation, Formal Analysis, Investigation, Methodology, Project Administration, Resources, Supervision, Validation, Visualization, writing – Original Draft Preparation, Writing – Review and Editing. NS: Formal Analysis, Review and Editing. AM: Review and Editing. AR: Review and Editing. AG: Review and Editing. All authors contributed to the article and approved the submitted version.

## Conflict of Interest

The authors declare that the research was conducted in the absence of any commercial or financial relationships that could be construed as a potential conflict of interest.

## Supplementary Material

Supplementary File 1 Contains the information about all samples in all datasets.

Supplementary File 2 Contains the quality plots.

Supplementary File 3 Contains all DEGs between all pairwise groups of samples.

Supplementary File 4 Contains network centralities.

Supplementary File 5 Contains enrichment information.

Supplementary File 6 Contains survival plots.
