# Supplementary figures and images for: Systems Biomedicine of Primary and Metastatic Colorectal Cancer Reveals Potential Therapeutic Targets
Source: Front Oncol. 2021 Jun 24;11:597536. doi: 10.3389/fonc.2021.597536 (PMC8263939; doi:10.3389/fonc.2021.597536)

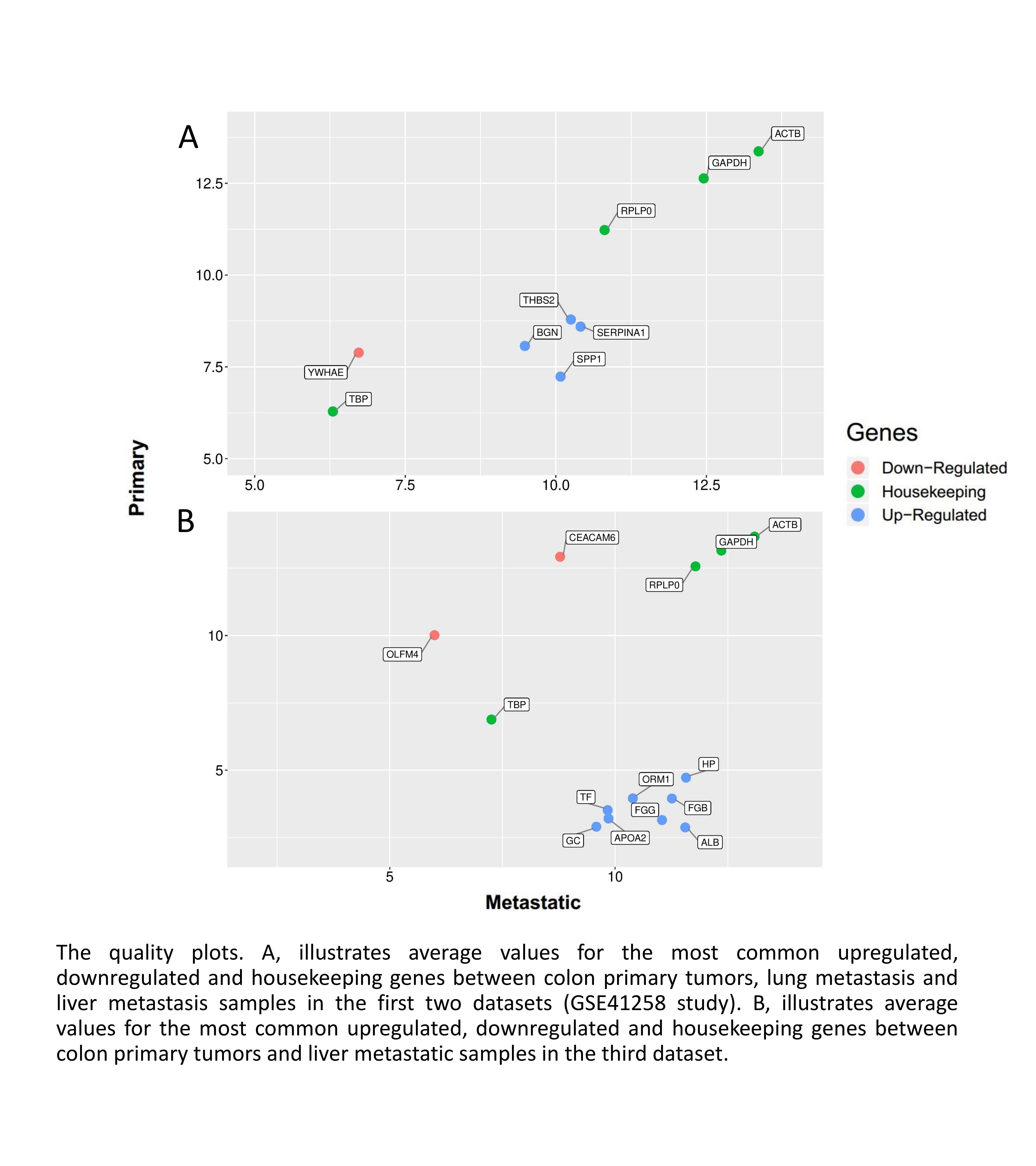

Supplement: Supplementary File 2 — Contains the quality plots. [file DataSheet_2.zip › Supplementary file 2.JPEG]

# Overall Survival

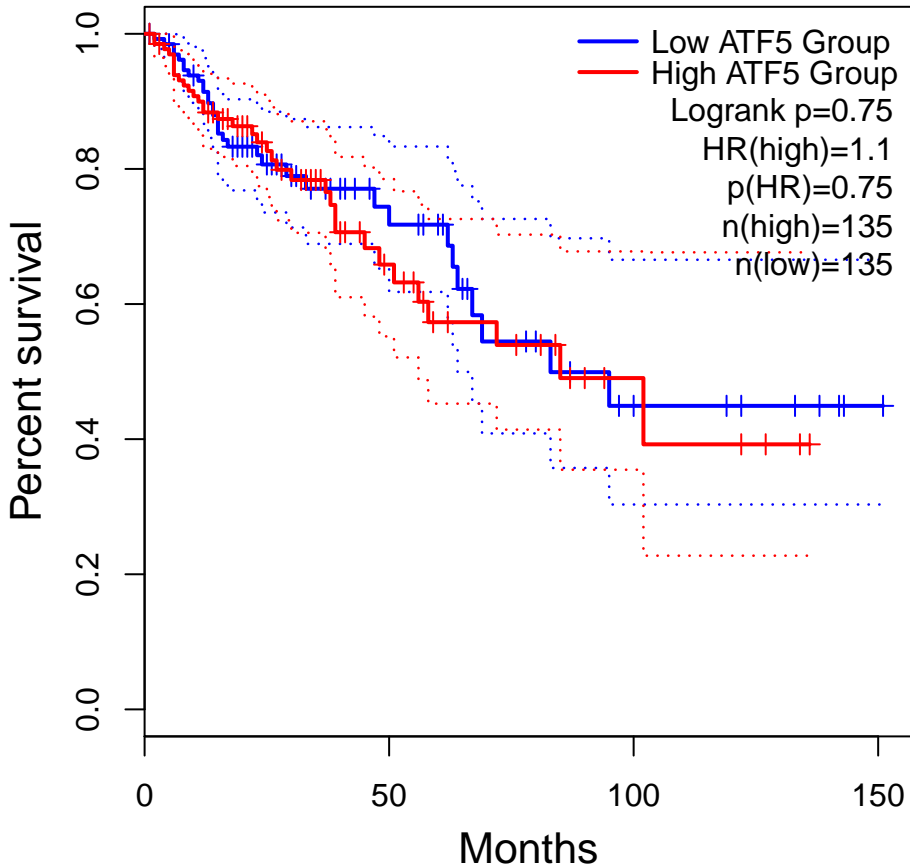

Supplement: Supplementary File 6 — Contains survival plots. [file DataSheet_6.zip › Supplementary file 6/ATF5_survival_SP6tB.pdf]

# Overall Survival

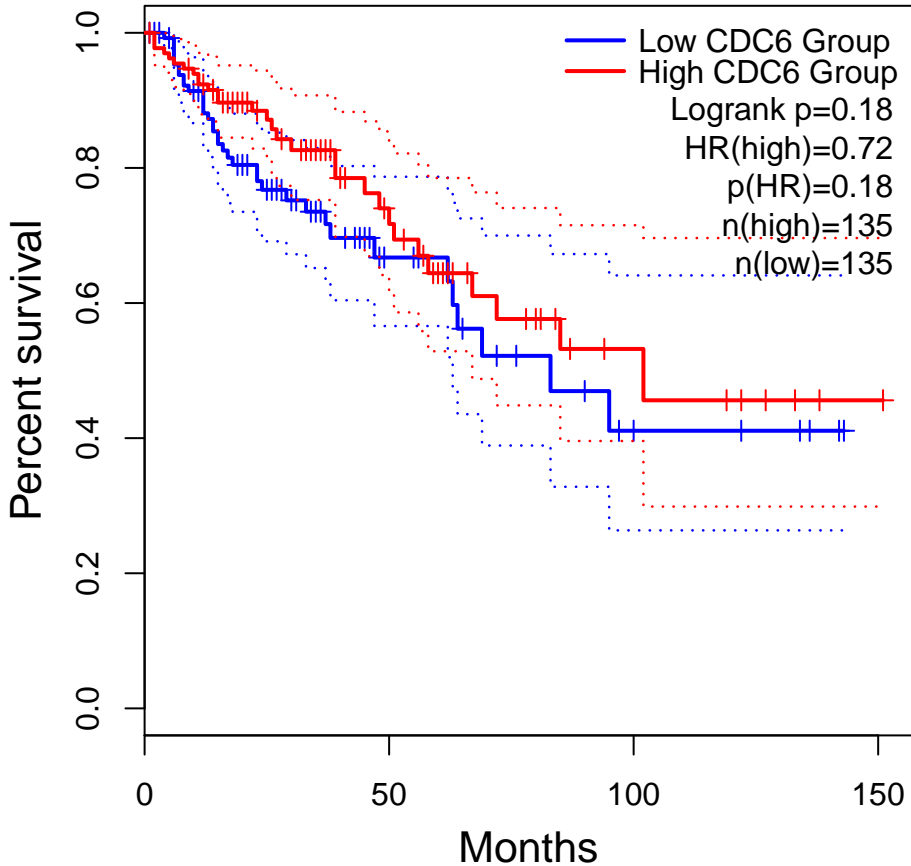

Supplement: Supplementary File 6 — Contains survival plots. [file DataSheet_6.zip › Supplementary file 6/CDC6_survival_rRgMi.pdf]

# Overall Survival

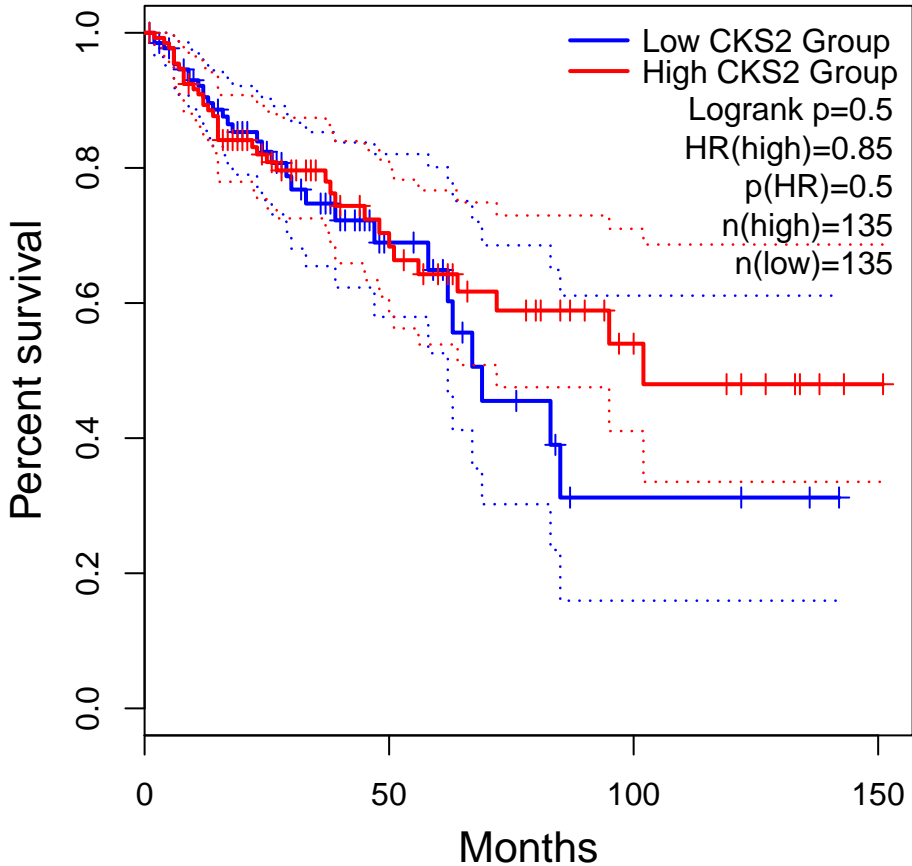

Supplement: Supplementary File 6 — Contains survival plots. [file DataSheet_6.zip › Supplementary file 6/CKS2_survival_mfgwl.pdf]

# Overall Survival

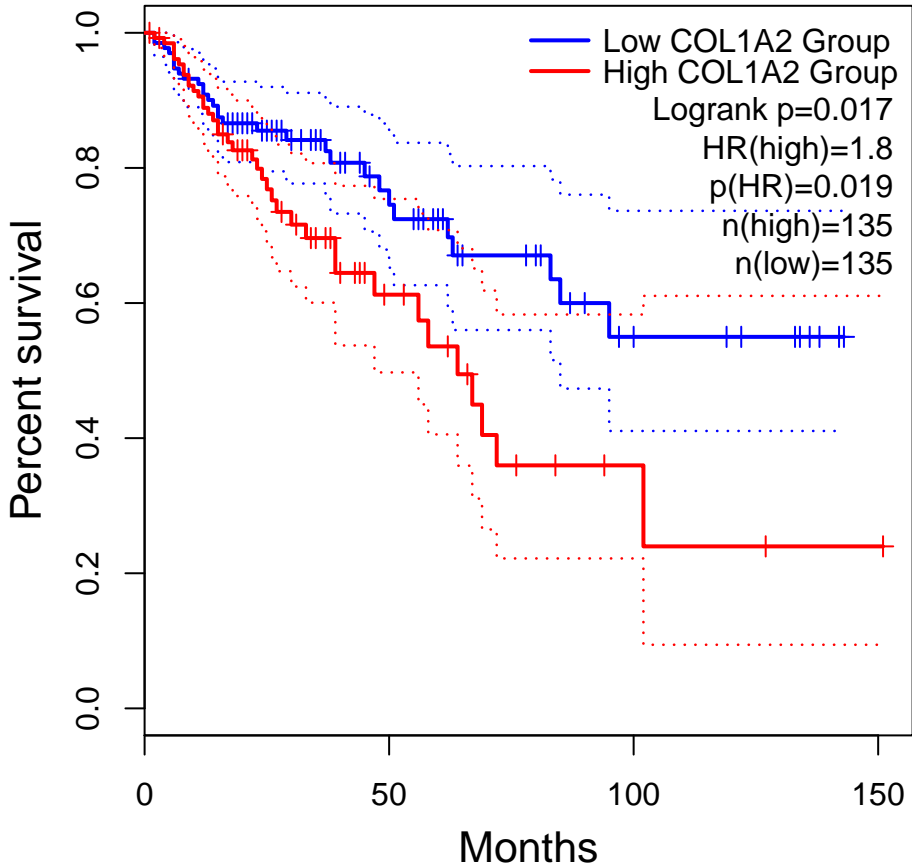

Supplement: Supplementary File 6 — Contains survival plots. [file DataSheet_6.zip › Supplementary file 6/COL1A2_survival_SBM79.pdf]

# Overall Survival

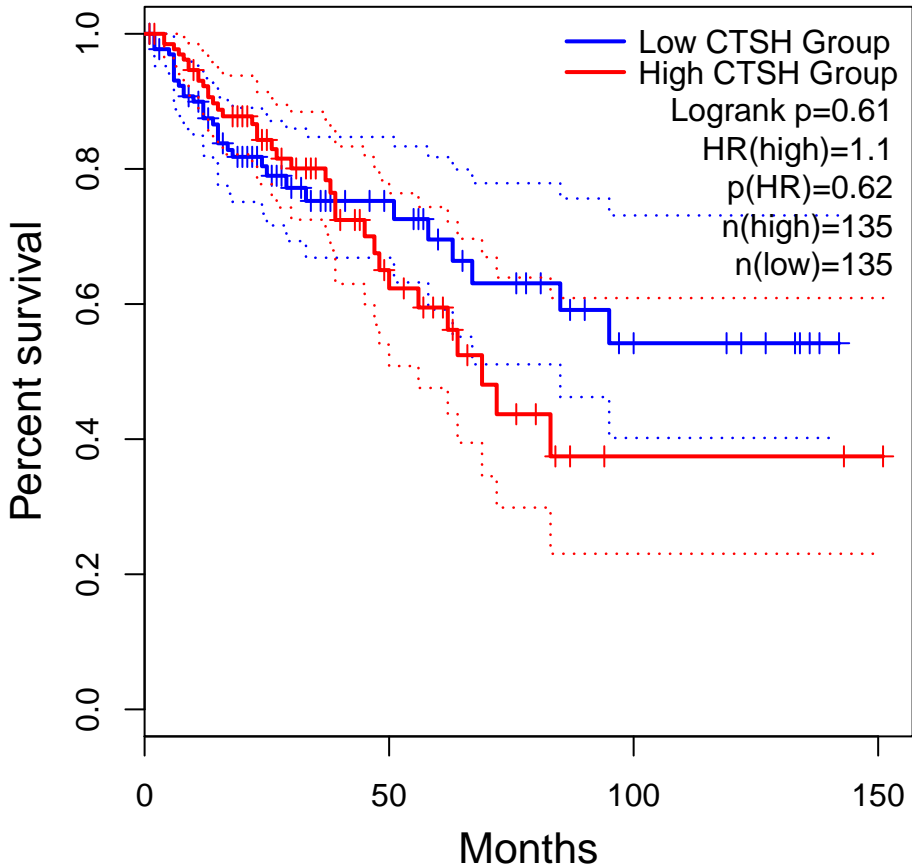

Supplement: Supplementary File 6 — Contains survival plots. [file DataSheet_6.zip › Supplementary file 6/CTSH_survival_mxNdj.pdf]

# Overall Survival

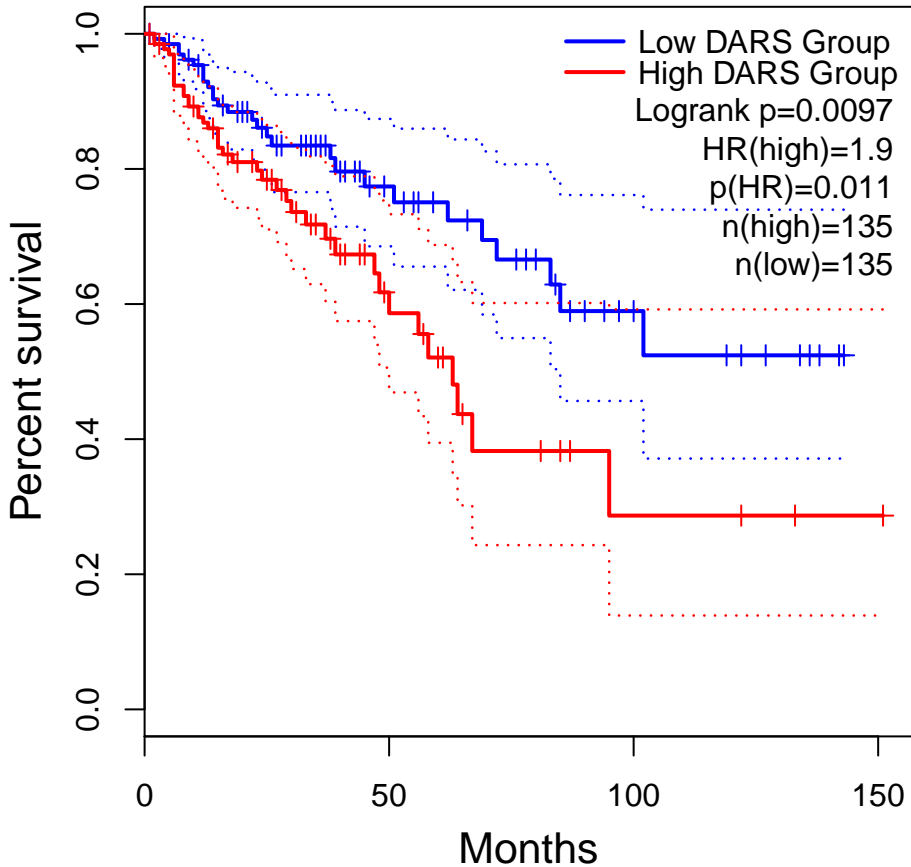

Supplement: Supplementary File 6 — Contains survival plots. [file DataSheet_6.zip › Supplementary file 6/DARS_survival_wnrLa.pdf]

# Overall Survival

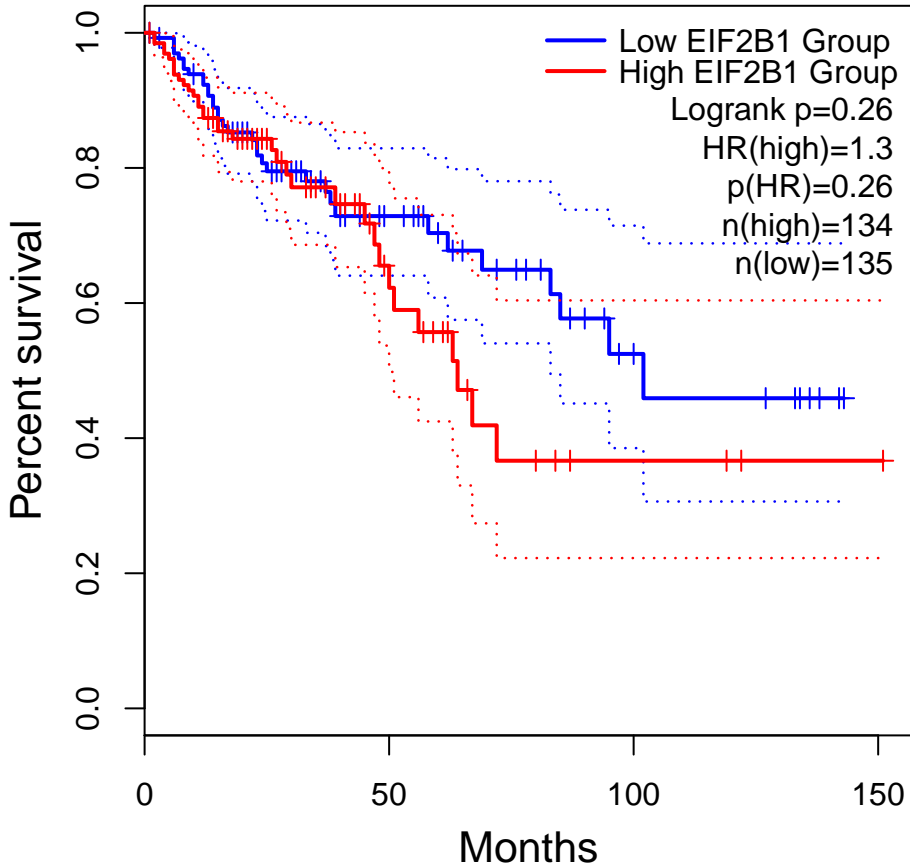

Supplement: Supplementary File 6 — Contains survival plots. [file DataSheet_6.zip › Supplementary file 6/EIF2B1_survival_Wg05Q.pdf]

# Overall Survival

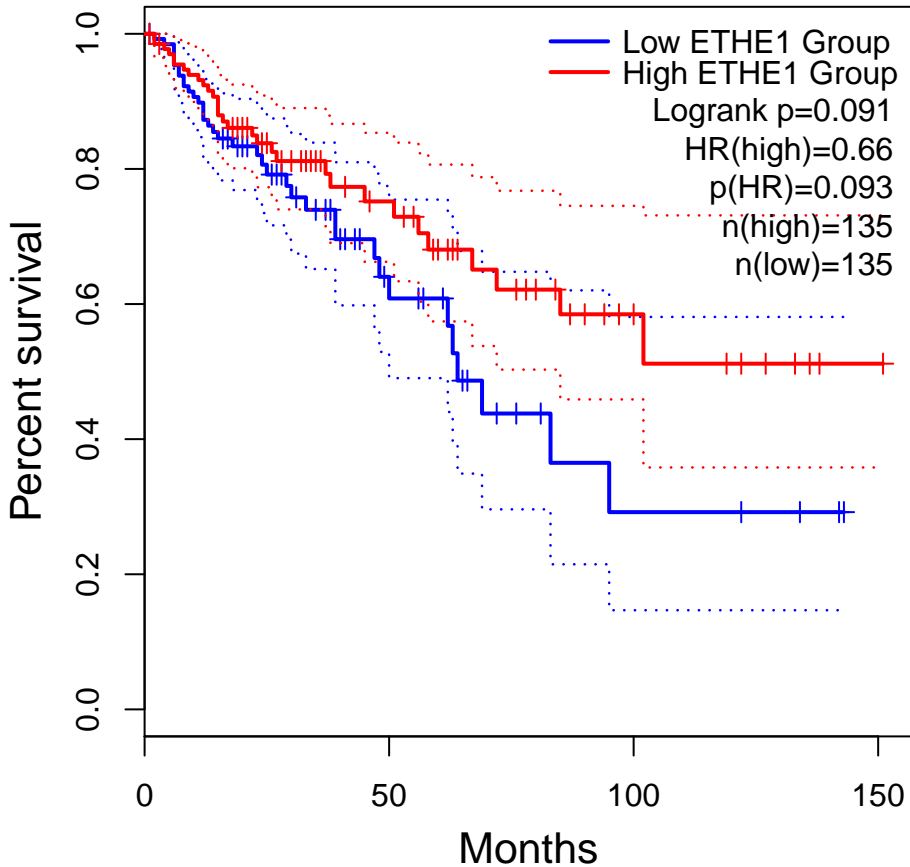

Supplement: Supplementary File 6 — Contains survival plots. [file DataSheet_6.zip › Supplementary file 6/ETHE1_survival_8WQ9W.pdf]

# Overall Survival

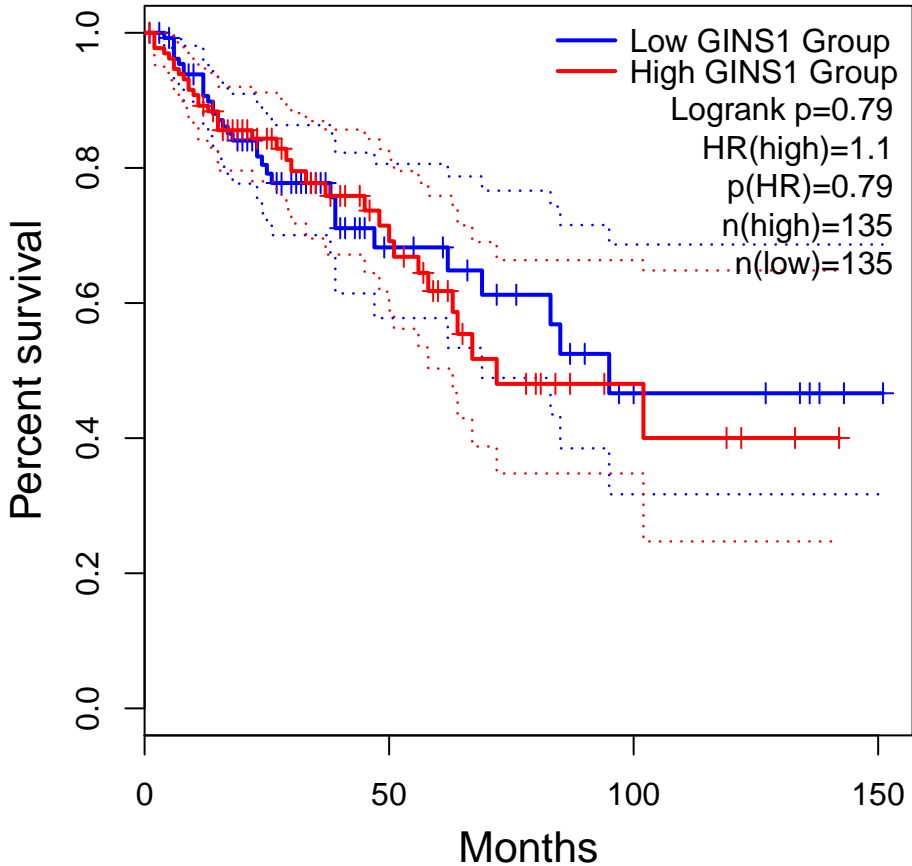

Supplement: Supplementary File 6 — Contains survival plots. [file DataSheet_6.zip › Supplementary file 6/GINS1_survival_w8vdh.pdf]

# Overall Survival

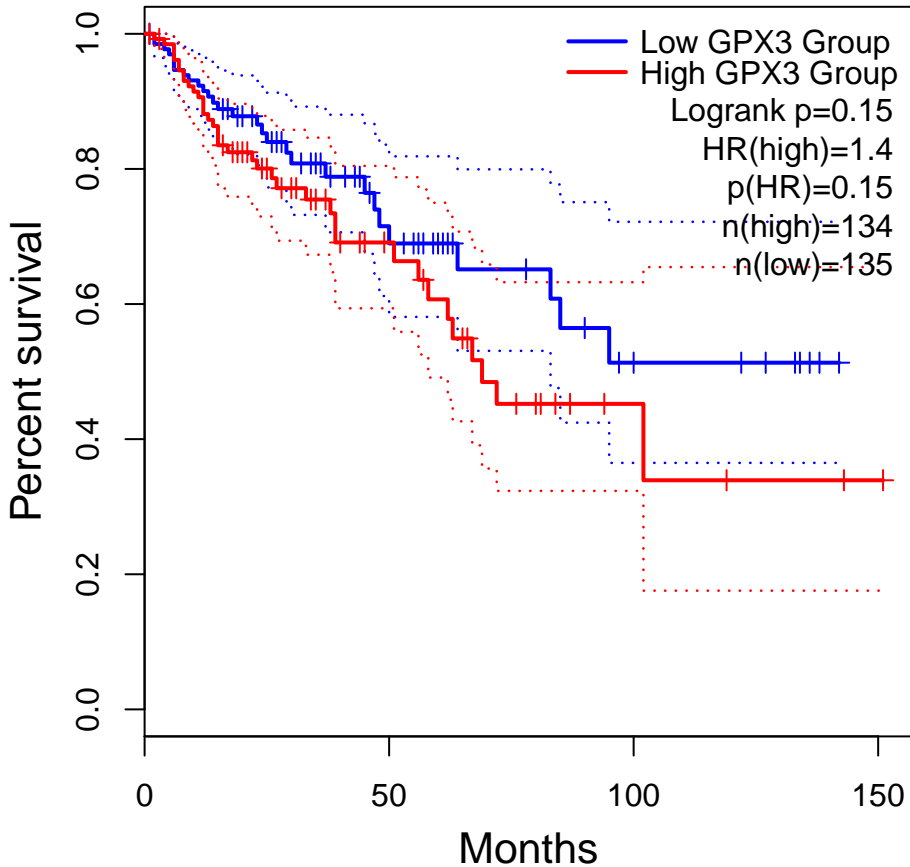

Supplement: Supplementary File 6 — Contains survival plots. [file DataSheet_6.zip › Supplementary file 6/GPX3_survival_5h9Di.pdf]

# Overall Survival

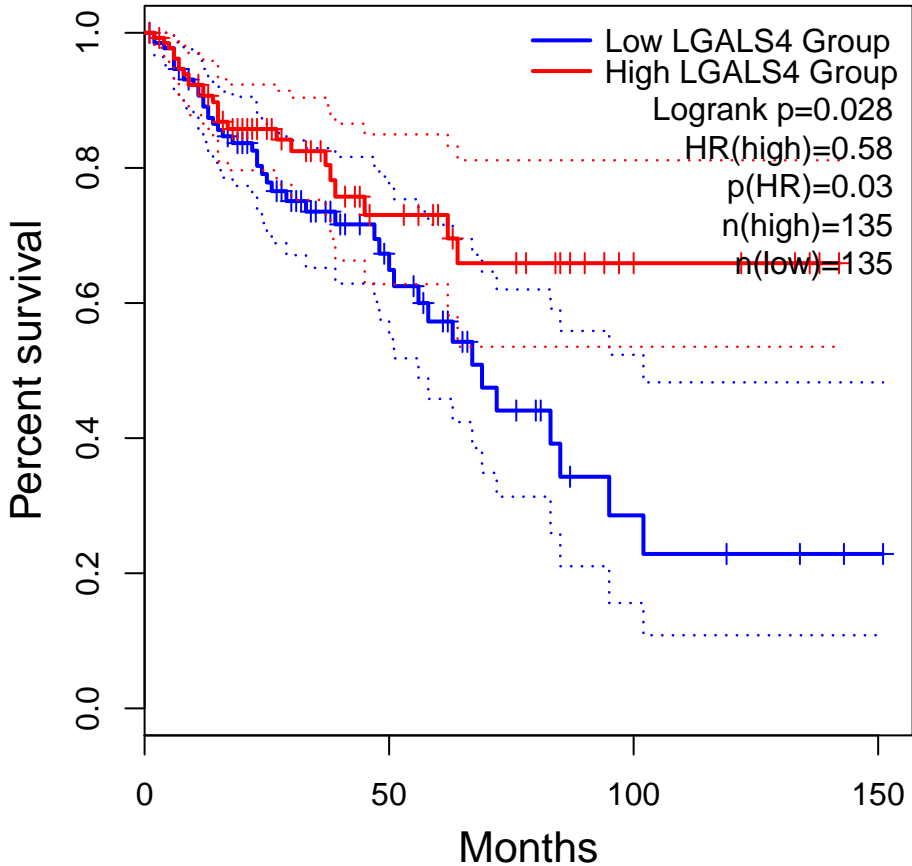

Supplement: Supplementary File 6 — Contains survival plots. [file DataSheet_6.zip › Supplementary file 6/LGALS4_survival_Aj2KE.pdf]

# Overall Survival

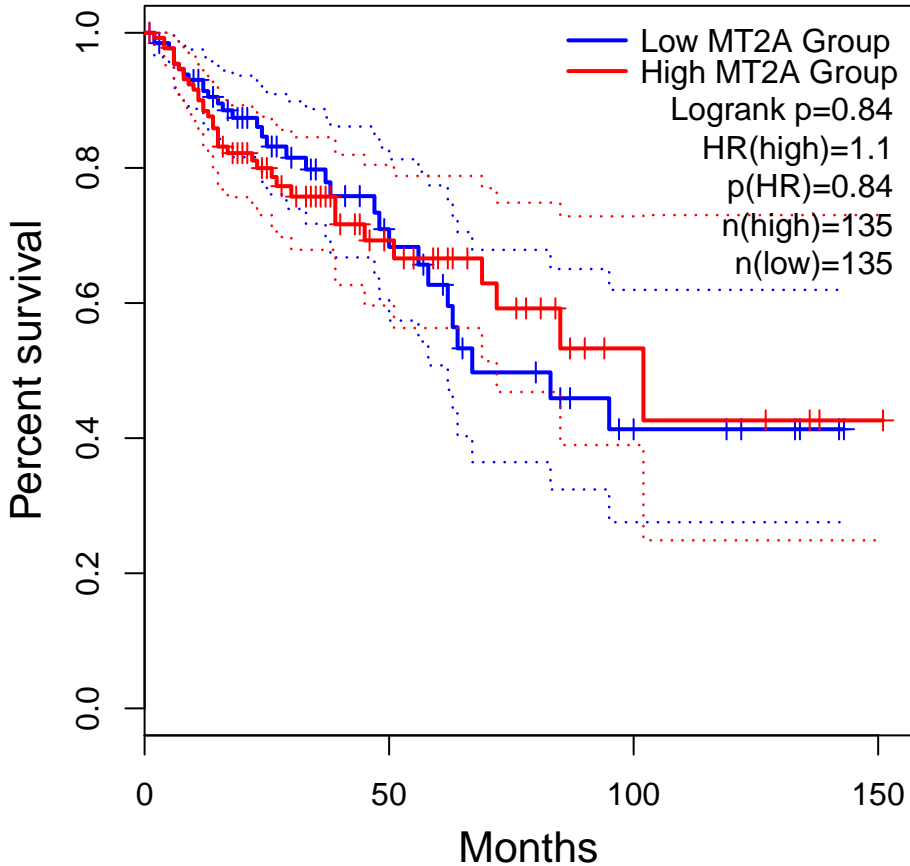

Supplement: Supplementary File 6 — Contains survival plots. [file DataSheet_6.zip › Supplementary file 6/MT2A_survival_0BxfJ.pdf]

# Overall Survival

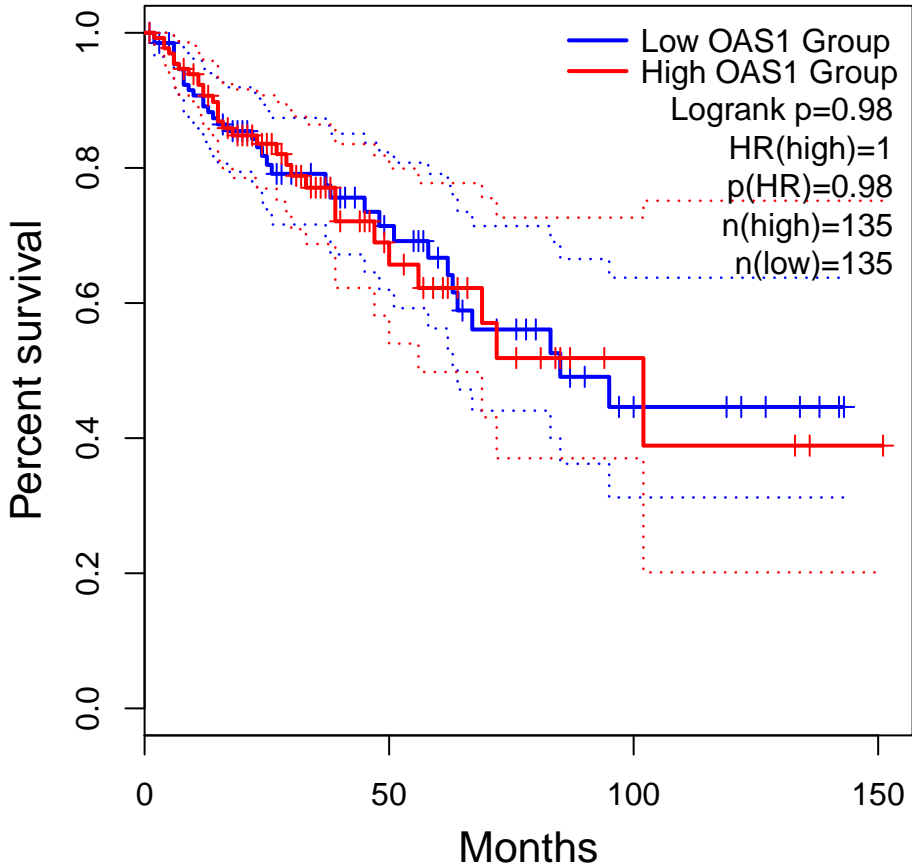

Supplement: Supplementary File 6 — Contains survival plots. [file DataSheet_6.zip › Supplementary file 6/OAS1_survival_8wPL6.pdf]

# Overall Survival

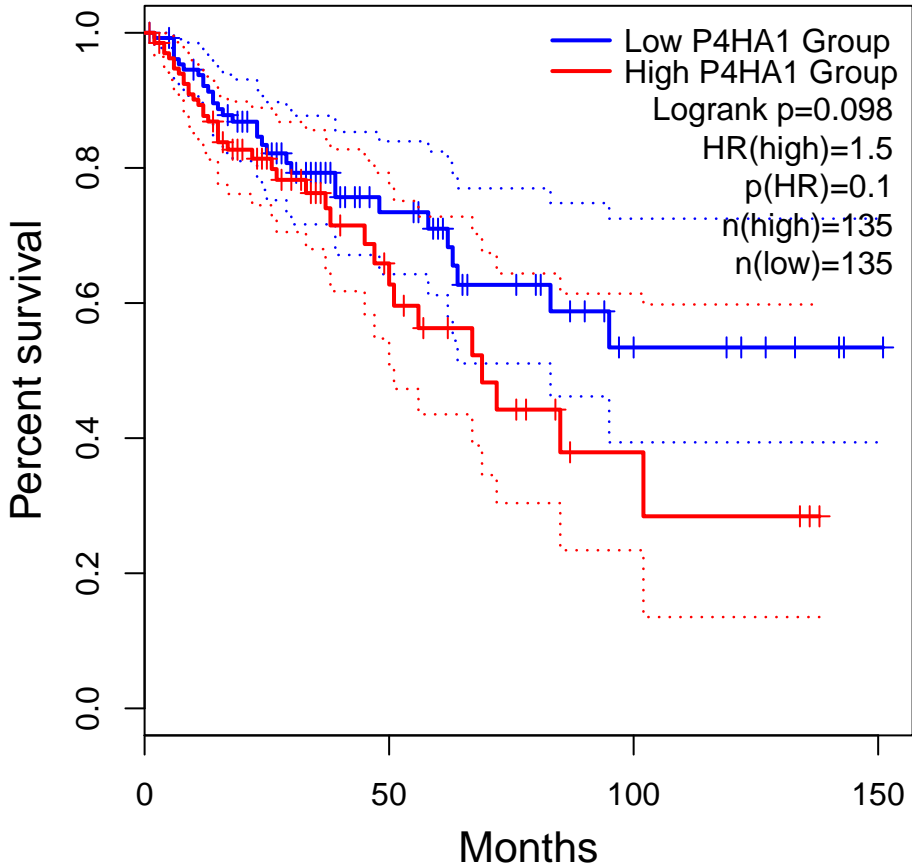

Supplement: Supplementary File 6 — Contains survival plots. [file DataSheet_6.zip › Supplementary file 6/P4HA1_survival_nrBt0.pdf]

# Overall Survival

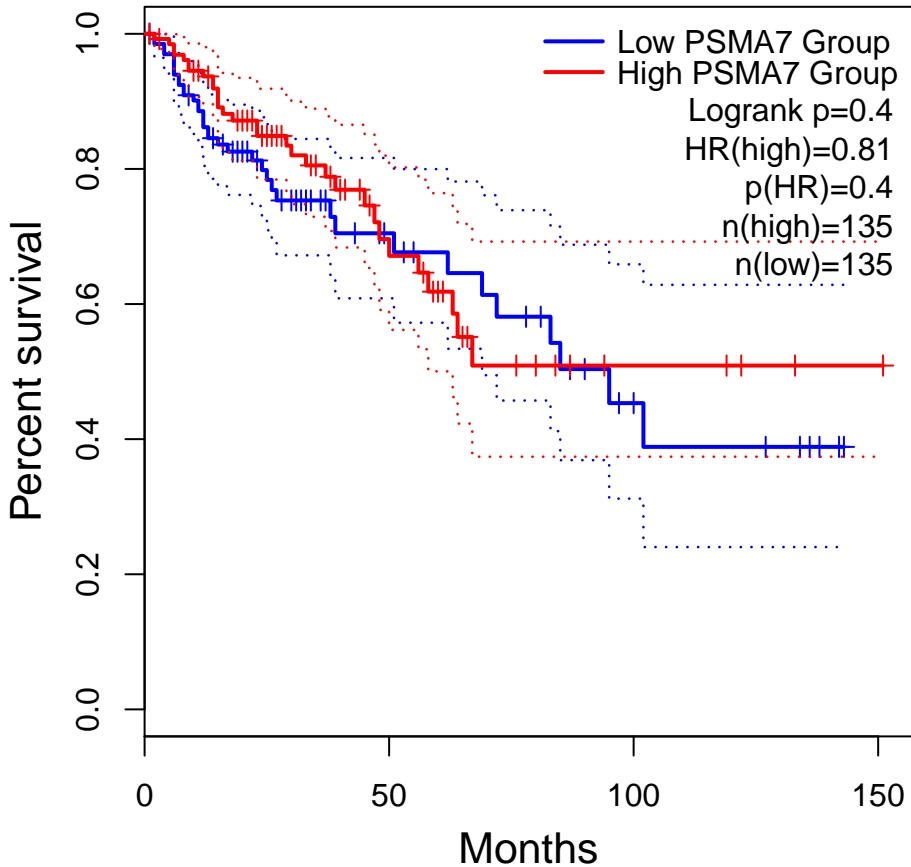

Supplement: Supplementary File 6 — Contains survival plots. [file DataSheet_6.zip › Supplementary file 6/PSMA7_survival_AMf4g.pdf]

# Overall Survival

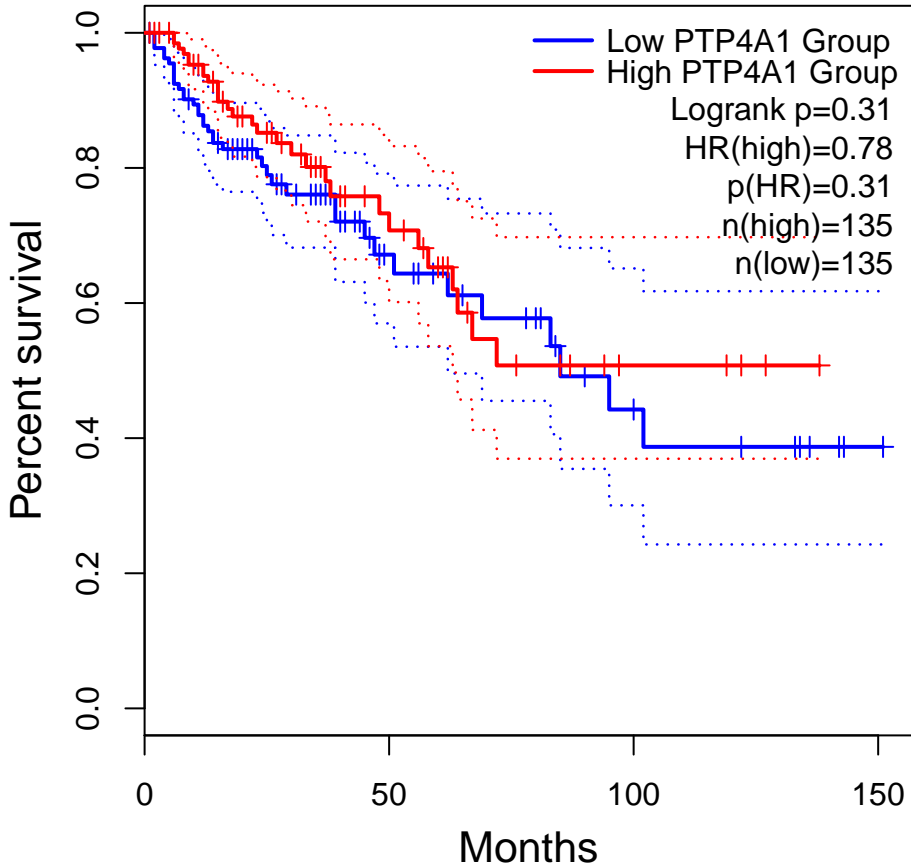

Supplement: Supplementary File 6 — Contains survival plots. [file DataSheet_6.zip › Supplementary file 6/PTP4A1_survival_edrpF.pdf]

# Overall Survival

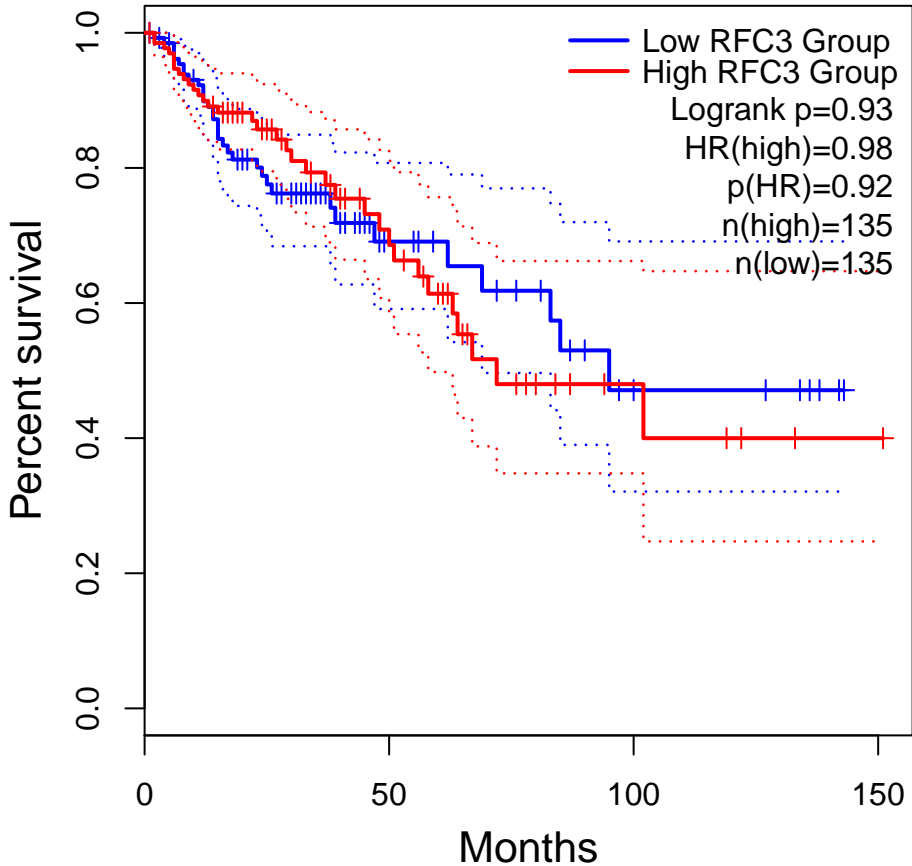

Supplement: Supplementary File 6 — Contains survival plots. [file DataSheet_6.zip › Supplementary file 6/RFC3_survival_KOO7E.pdf]

# Overall Survival

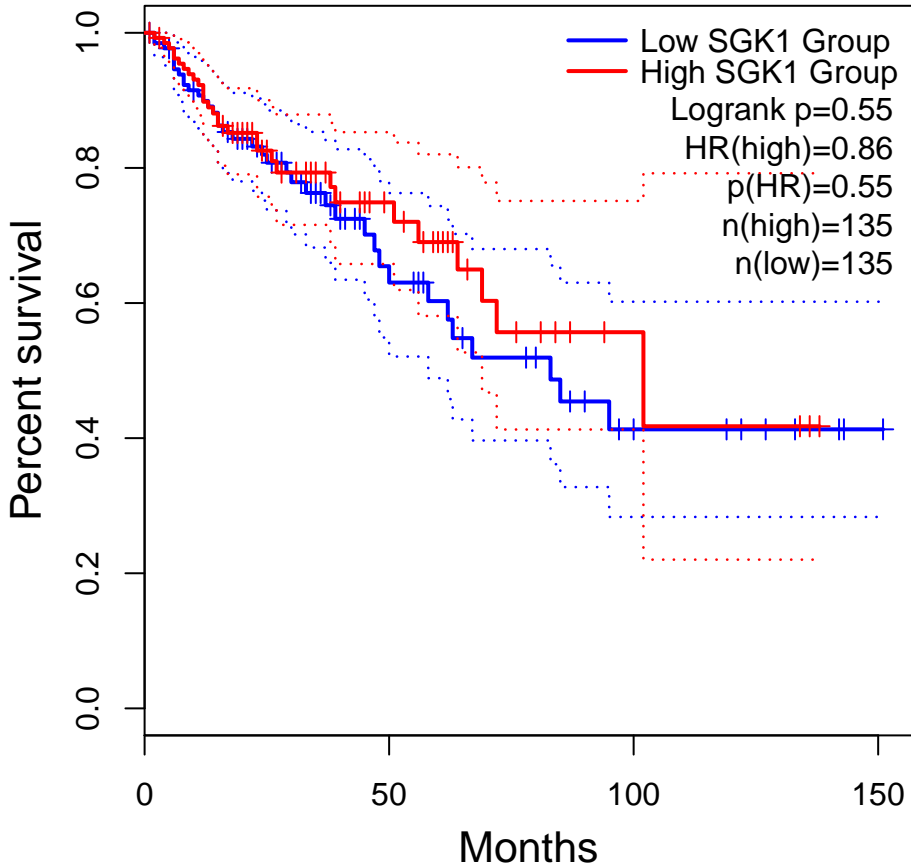

Supplement: Supplementary File 6 — Contains survival plots. [file DataSheet_6.zip › Supplementary file 6/SGK1_survival_05RYj.pdf]

# Overall Survival

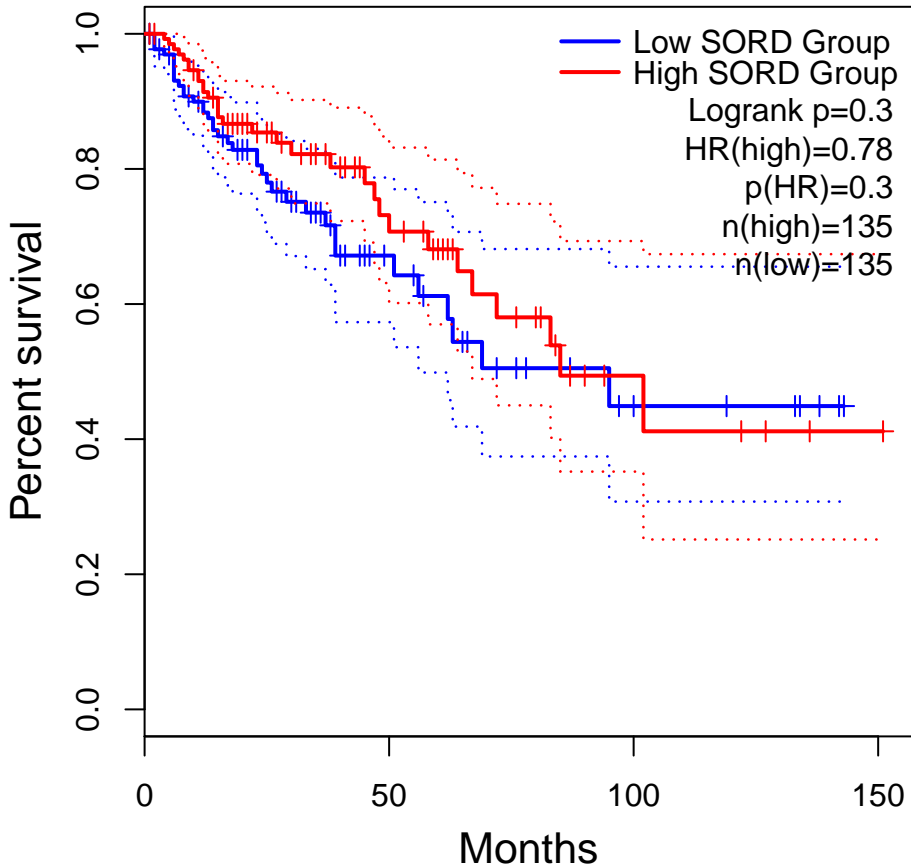

Supplement: Supplementary File 6 — Contains survival plots. [file DataSheet_6.zip › Supplementary file 6/SORD_survival_dKN9M.pdf]

# Overall Survival

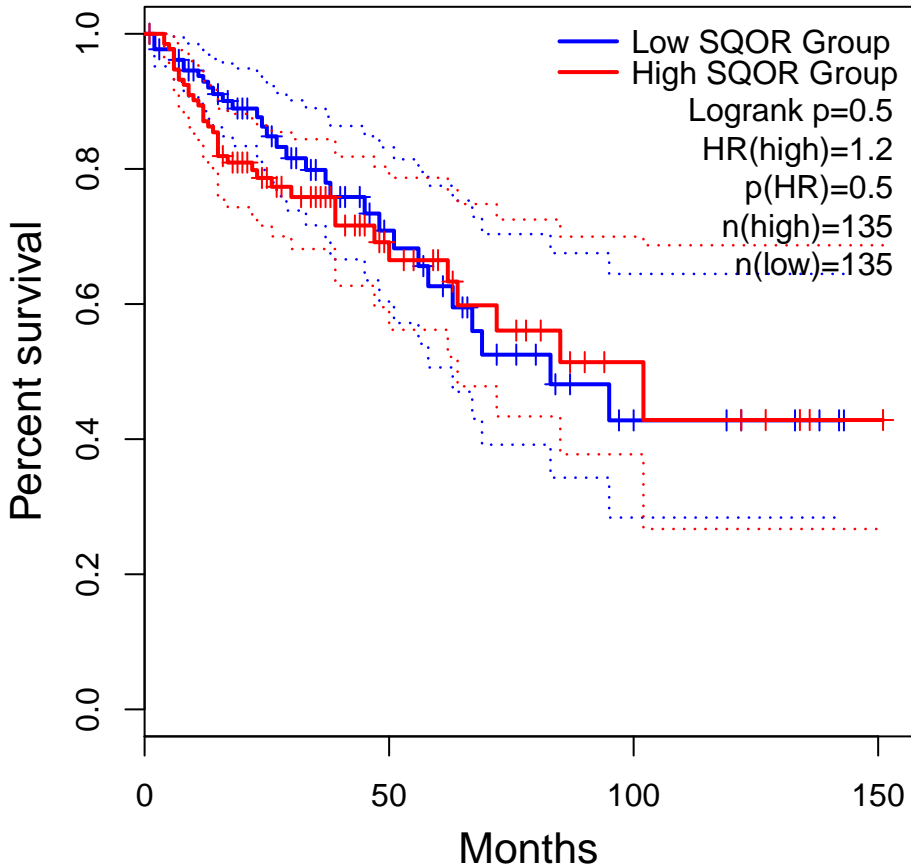

Supplement: Supplementary File 6 — Contains survival plots. [file DataSheet_6.zip › Supplementary file 6/SQOR_survival_Cn3yD.pdf]

# Overall Survival

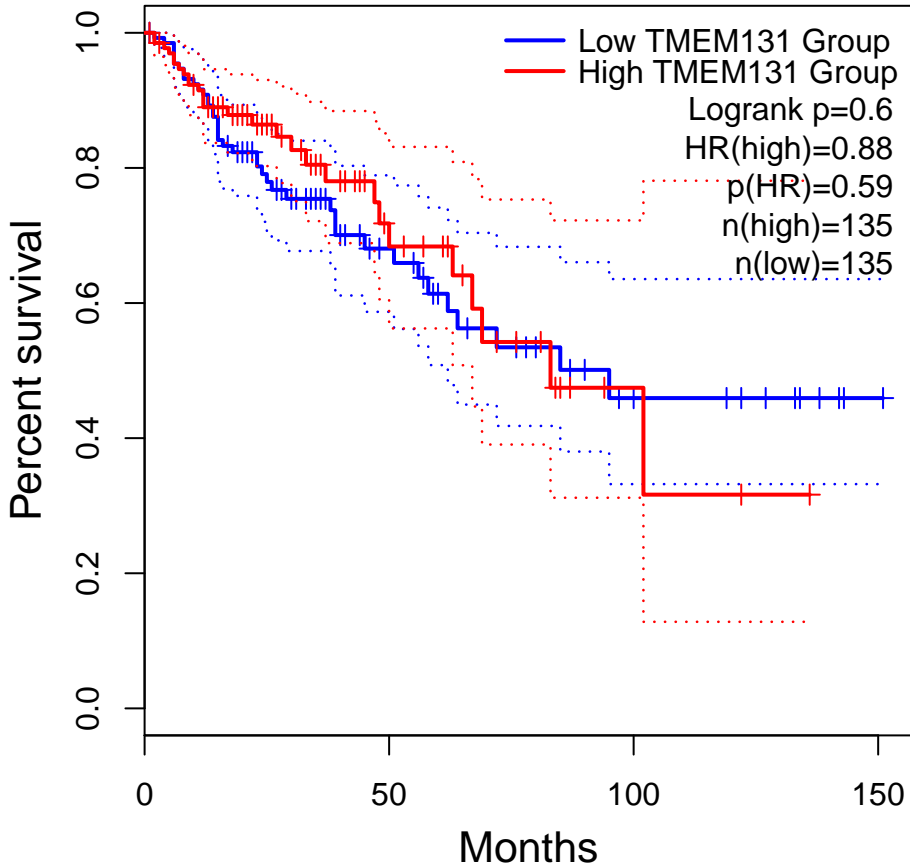

Supplement: Supplementary File 6 — Contains survival plots. [file DataSheet_6.zip › Supplementary file 6/TMEM131_survival_os5QZ.pdf]

# Overall Survival

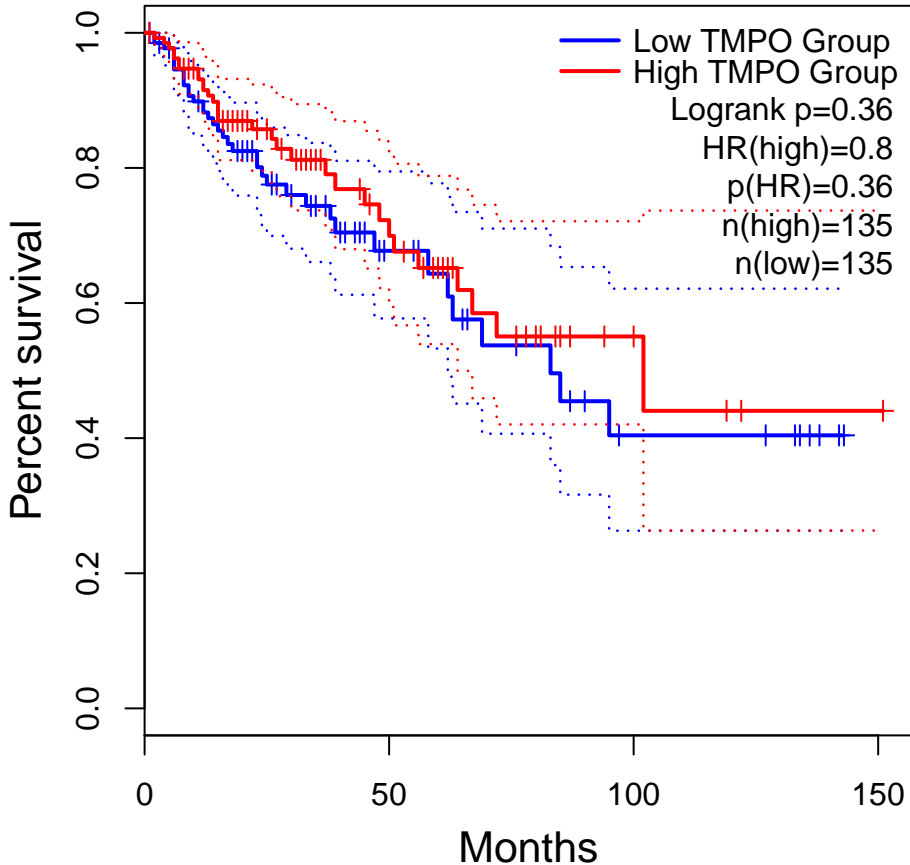

Supplement: Supplementary File 6 — Contains survival plots. [file DataSheet_6.zip › Supplementary file 6/TMPO_survival_5I1kS.pdf]

# Overall Survival

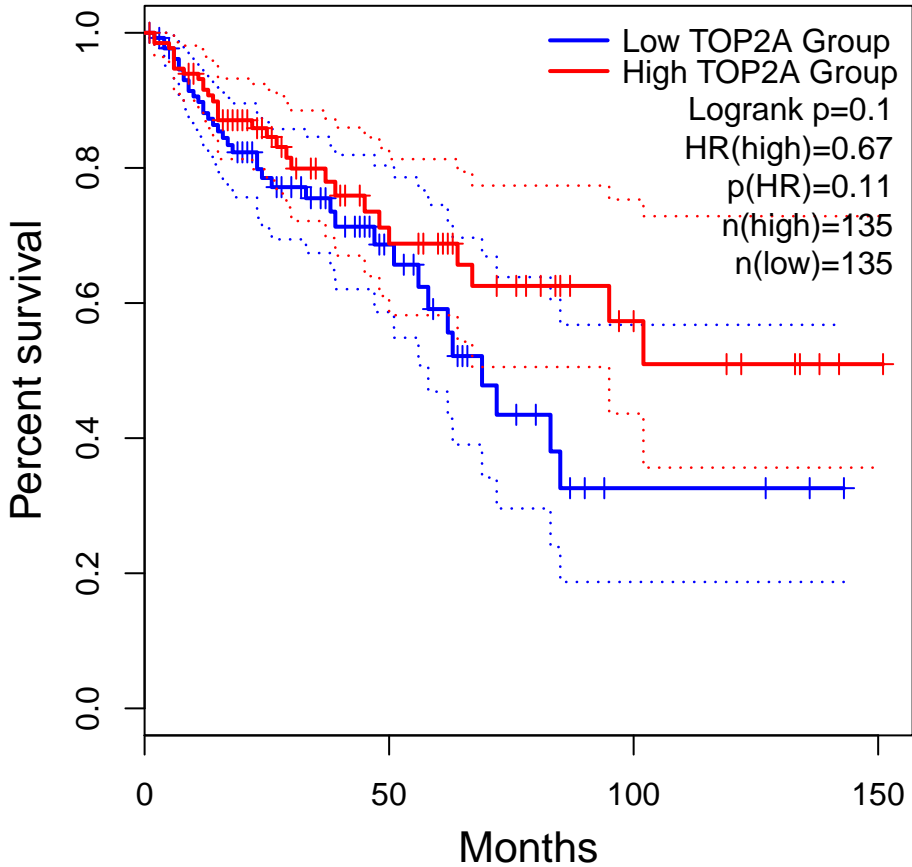

Supplement: Supplementary File 6 — Contains survival plots. [file DataSheet_6.zip › Supplementary file 6/TOP2A_survival_R2tI2.pdf]

# Overall Survival

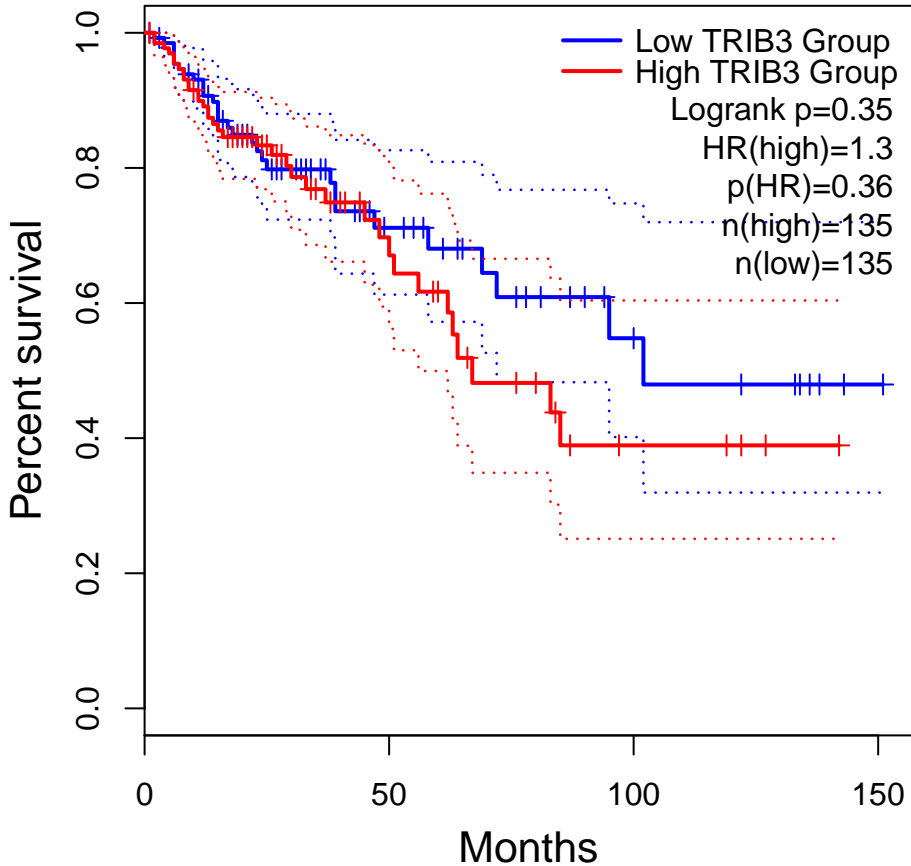

Supplement: Supplementary File 6 — Contains survival plots. [file DataSheet_6.zip › Supplementary file 6/TRIB3_survival_lE7SY.pdf]

# Overall Survival

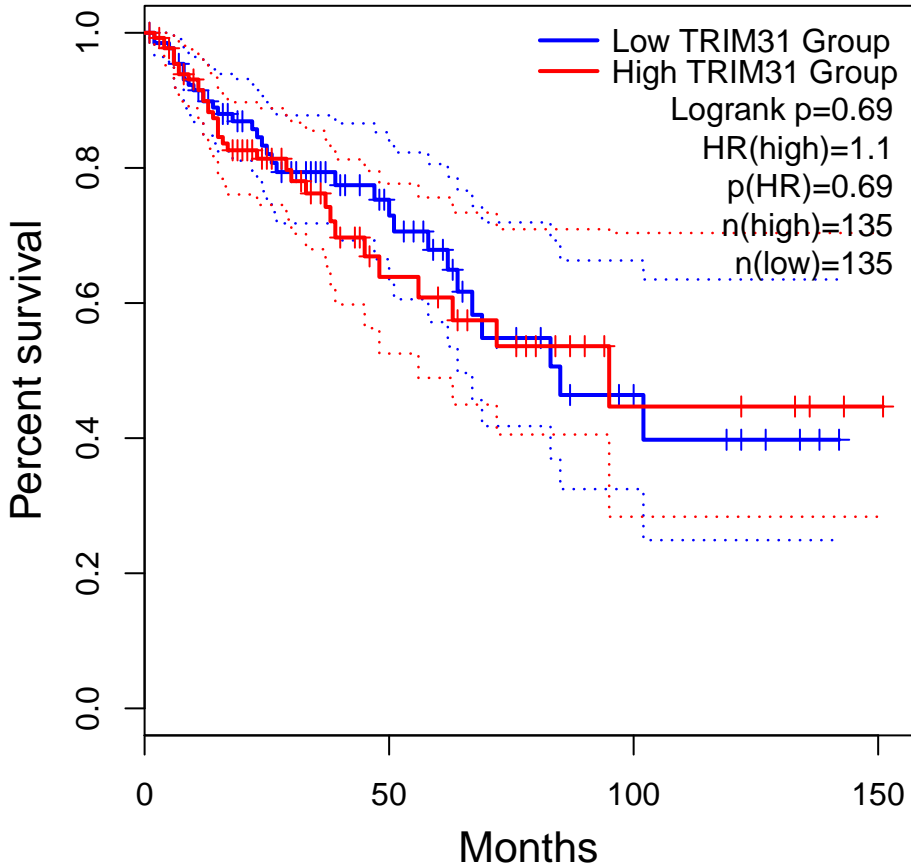

Supplement: Supplementary File 6 — Contains survival plots. [file DataSheet_6.zip › Supplementary file 6/TRIM31_survival_kVrff.pdf]

# Overall Survival

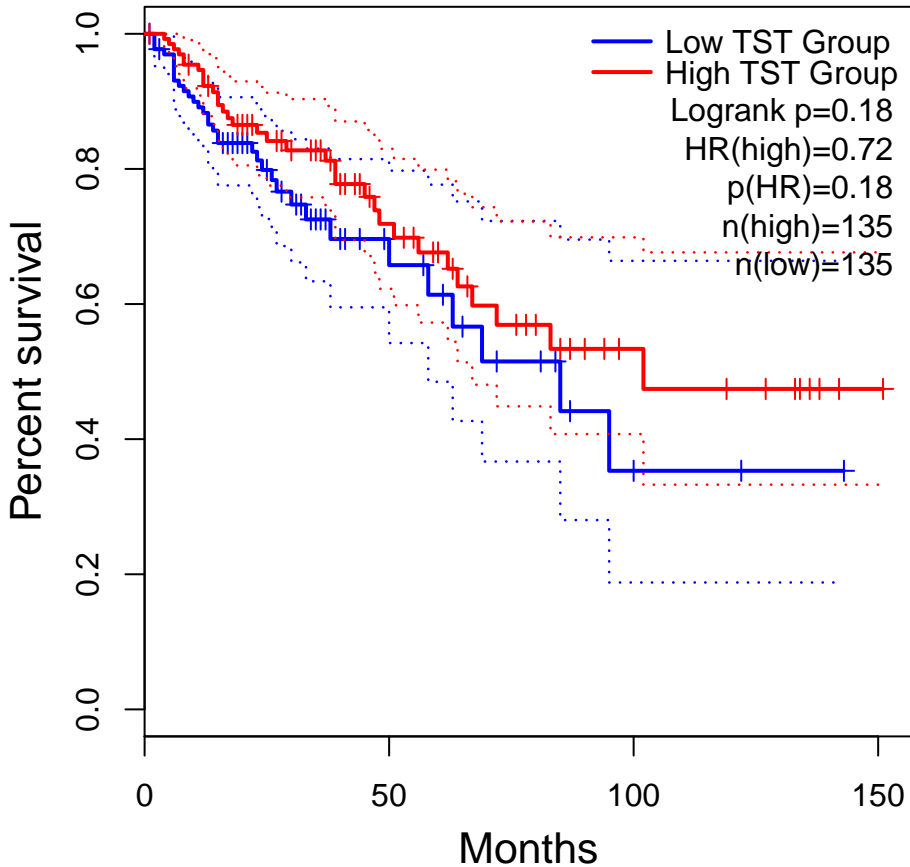

Supplement: Supplementary File 6 — Contains survival plots. [file DataSheet_6.zip › Supplementary file 6/TST_survival_vactu.pdf]

# Overall Survival

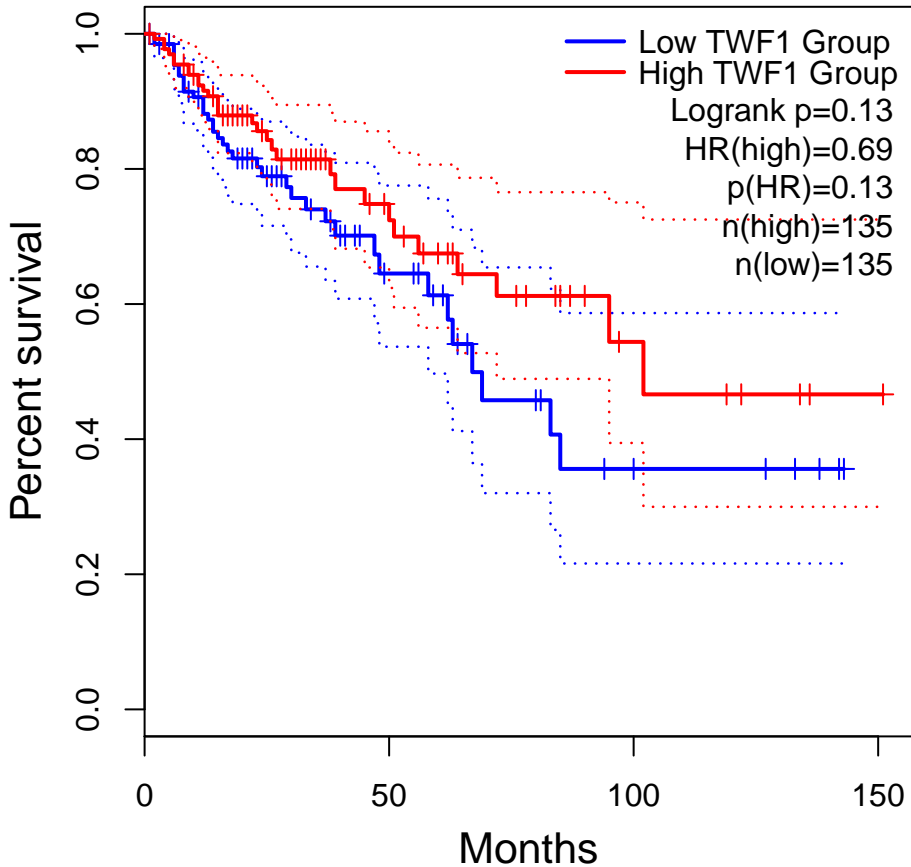

Supplement: Supplementary File 6 — Contains survival plots. [file DataSheet_6.zip › Supplementary file 6/TWF1_survival_rR1i0.pdf]
