# Supplementary material for: Systems Biomedicine of Primary and Metastatic Colorectal Cancer Reveals Potential Therapeutic Targets
Source: Front Oncol. 2021 Jun 24;11:597536. doi: 10.3389/fonc.2021.597536 (PMC8263939; doi:10.3389/fonc.2021.597536)
Supplement: Supplementary File 6 — Contains survival plots. [file DataSheet_6.zip › Supplementary file 6/survival_map_5Y69H.pdf]

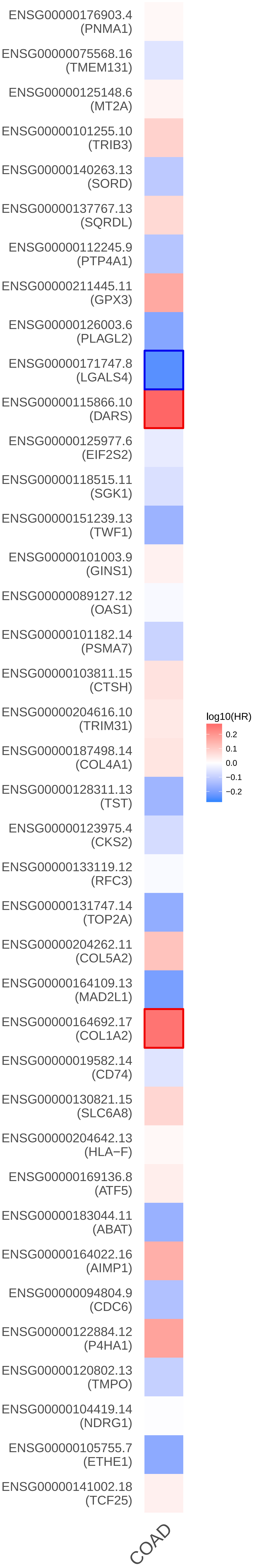

ENSG00000176903.4 (PNMA1)
ENSG00000075568.16 (TMEM131)
ENSG00000125148.6 (MT2A)
ENSG00000101255.10 (TRIB3)
ENSG00000140263.13 (SORD)
ENSG00000137767.13 (SQRDL)
ENSG00000112245.9 (PTP4A1)
ENSG00000211445.11 (GPX3)
ENSG00000126003.6 (PLAGL2)
ENSG00000171747.8 (LGALS4)
ENSG00000115866.10 (DARS)
ENSG00000125977.6 (EIF2S2)
ENSG00000118515.11 (SGK1)
ENSG00000151239.13 (TWF1)
ENSG00000101003.9 (GINS1)
ENSG00000089127.12 (OAS1)
ENSG00000101182.14 (PSMA7)
ENSG00000103811.15 (CTSH)
ENSG00000204616.10 (TRIM31)
ENSG00000187498.14 (COL4A1)
ENSG00000128311.13 (TST)
ENSG00000123975.4 (CKS2)
ENSG00000133119.12 (RFC3)
ENSG00000131747.14 (TOP2A)
ENSG00000204262.11 (COL5A2)
ENSG00000164109.13 (MAD2L1)
ENSG00000164692.17 (COL1A2)
ENSG00000019582.14 (CD74)
ENSG00000130821.15 (SLC6A8)
ENSG00000204642.13 (HLA−F)
ENSG00000169136.8 (ATF5)
ENSG00000183044.11 (ABAT)
ENSG00000164022.16 (AIMP1)
ENSG00000094804.9 (CDC6)
ENSG00000122884.12 (P4HA1)
ENSG00000120802.13 (TMPO)
ENSG00000104419.14 (NDRG1)
ENSG00000105755.7 (ETHE1)
ENSG00000141002.18 (TCF25)
COAD
log10(HR)
0.2
0.1
0.0
−0.1
−0.2
